# Single-cell transcriptional profiles in human skeletal muscle

**DOI:** 10.1038/s41598-019-57110-6

**Published:** 2020-01-14

**Authors:** Aliza B. Rubenstein, Gregory R. Smith, Ulrika Raue, Gwénaëlle Begue, Kiril Minchev, Frederique Ruf-Zamojski, Venugopalan D. Nair, Xingyu Wang, Lan Zhou, Elena Zaslavsky, Todd A. Trappe, Scott Trappe, Stuart C. Sealfon

**Affiliations:** 1 Department of Neurology, Icahn School of Medicine at Mount Sinai, New York, New York, 10029 USA; 2 Center for Advanced Research on Diagnostic Assays (CARDA), Icahn School of Medicine at Mount Sinai, New York, New York, 10029 USA; 3 Human Performance Laboratory, Ball State University, Muncie, Indiana, 47306 USA; 4 Department of Neurology, Boston University Medical Center, Boston, MA 02118 USA

**Keywords:** Functional clustering, Transcriptomics, Physiology

## Abstract

Skeletal muscle is a heterogeneous tissue comprised of muscle fiber and mononuclear cell types that, in addition to movement, influences immunity, metabolism and cognition. We investigated the gene expression patterns of skeletal muscle cells using RNA-seq of subtype-pooled single human muscle fibers and single cell RNA-seq of mononuclear cells from human vastus lateralis, mouse quadriceps, and mouse diaphragm. We identified 11 human skeletal muscle mononuclear cell types, including two fibro-adipogenic progenitor (FAP) cell subtypes. The human FBN1+ FAP cell subtype is novel and a corresponding FBN1+ FAP cell type was also found in single cell RNA-seq analysis in mouse. Transcriptome exercise studies using bulk tissue analysis do not resolve changes in individual cell-type proportion or gene expression. The cell-type gene signatures provide the means to use computational methods to identify cell-type level changes in bulk studies. As an example, we analyzed public transcriptome data from an exercise training study and revealed significant changes in specific mononuclear cell-type proportions related to age, sex, acute exercise and training. Our single-cell expression map of skeletal muscle cell types will further the understanding of the diverse effects of exercise and the pathophysiology of muscle disease.

## Introduction

Skeletal muscle is a complex heterogeneous tissue consisting of multinucleated muscle fibers, immune cells, endothelial cells, muscle stem cells (satellite cells), non-myogenic mesenchymal progenitors (e.g., fibro-adipogenic progenitors, or FAPs), and other mononuclear cells^1^. To improve the understanding of skeletal muscle cell types and their transcriptional signatures, we studied human and mouse skeletal muscle mononuclear cells by single-cell RNA-sequencing and single human muscle fiber subtypes by RNA-seq.

The majority of skeletal muscle is composed of the multinucleated fibers that facilitate movement. These muscle fibers include several fiber types of differing metabolic and functional properties^2–4^. While slow-twitch (or Type I) muscle fibers possess high oxidative capacity, fast-twitch (or Type II) muscle fibers have a high glycolytic capacity and are capable of supplying more power than Type I fibers^2–4^. Fiber-type composition differs across individuals and can change by as much as 10–30% during exercise training regimens^5–7^. Furthermore, the transcriptomic response to physical activity is different in each fiber-type as each fiber-type responds differently to different modes of exercise^8,9^. Crucially, muscle fibers secrete myokines, which both act locally within muscle tissue as well as influence other organs and tissues via hormone-like signaling^10^. Myokines may be responsible for the immune-, metabolism-, and cognition-related benefits of physical activity, as well as the chronic diseases that are caused by lack of physical activity (insulin resistance, cardiovascular disease, etc.)^10^.

Besides multinucleated fibers, skeletal muscle contains many mononuclear cells, such as immune cells, endothelial cells, satellite cells, and FAPs. Many of these cell populations are crucial for homeostasis and/or exercise response. For instance, immune cells promote inflammation in response to exercise and injury^1^, FAPs secrete IL−6, which promotes the differentiation of myogenic cells^11^, and endothelial cells secrete multiple myogenic and/or anti-apoptotic factors^12^. Due to the complex interplay between these cell types and their influence on muscle fibers, changes in cells within the skeletal muscle such as FAPs and satellite cells are implicated in degenerative muscle disease (e.g., muscular dystrophy, heterotopic ossification) as well as aging-related fibrosis and reduced muscle regeneration^1,13,14^.

Transcriptome studies of changes in skeletal muscle due to exercise, degenerative muscle disease, or other causes face the analysis challenge of distinguishing gene regulation within cells from changes in cell-type proportions, both of which cause an apparent regulation of the average expression in muscle of specific genes. To overcome this problem, accurate, experimentally-validated deconvolution methods have been developed that computationally estimate cell-type proportions in average transcriptome assays of mixed cell populations and allow changes in cell type proportion and changes in gene expression within cells to be distinguished^15,16^. However, no deconvolution methods have been applied to skeletal muscle tissue studies. Importantly, deconvolution methods require well-characterized gene signatures of individual cell types to estimate cell-type proportion.

Currently, there are few studies that have investigated the gene signatures of multinucleated and mononuclear cells in human skeletal muscle tissue. Previously, animal studies that examined gene expression differences between muscle fiber types did so by comparing muscle that is primarily Type I (soleus) with muscle that is primarily Type II (white quadriceps)^17^. While this study was able to determine several muscle type specific genes, it is unclear whether these genes are truly fiber type specific or simply differ between muscle locations. Previous transcriptome studies of manually dissected fiber-types analyzed mouse skeletal muscle^18,19^ or did not focus on human baseline transcriptome differences^8,20^. Isolated human skeletal muscle mononuclear cell populations (satellite cells, FAPs, etc.) as well as immune cells from blood samples have been studied previously^21–25^. However, only two recent studies have attempted to profile the skeletal muscle mononuclear cell population as a whole^26,27^, and both studies were performed on mouse skeletal muscle, not human.

To obtain comprehensive marker gene panels for both multinucleated fibers and mononuclear cells, we characterized the diverse cell types present in human skeletal muscle and developed cell-type gene signatures to deconvolve cell-type proportion and gene regulation within heterogeneous human skeletal muscle. We used RNA-seq on isolated muscle fibers and single-cell RNA-seq on mononuclear cells within skeletal muscle tissue using muscle single cell suspension to find a coherent gene signature for each cell type. We found several fiber-type marker genes that were not previously reported as fiber-type specific and discovered a hitherto uncharacterized human FAP cell subpopulation. Finally, to demonstrate the utility of these gene signatures in deconvolution of bulk skeletal muscle tissue, we used the cell-type signatures to deconvolve a previously published large human skeletal muscle microarray dataset^8^ and investigate changes in cell-type proportion. We found an increase in the endothelial/pericyte compartment due to exercise training, an increase in neutrophils in response to acute exercise, and a difference in lymphocyte proportion that is mediated by both age and gender. The cell-type gene signatures delineated here represent the first attempt to fully characterize the human skeletal muscle cell-type gene expression, the novel FAP subtype may elucidate skeletal muscle pathophysiologies, and the availability of these cell type signatures for deconvolution methods will enhance interpretation of human skeletal muscle transcriptome studies.

## Results

### Human mononuclear muscle cell signatures

**Figure 1:**
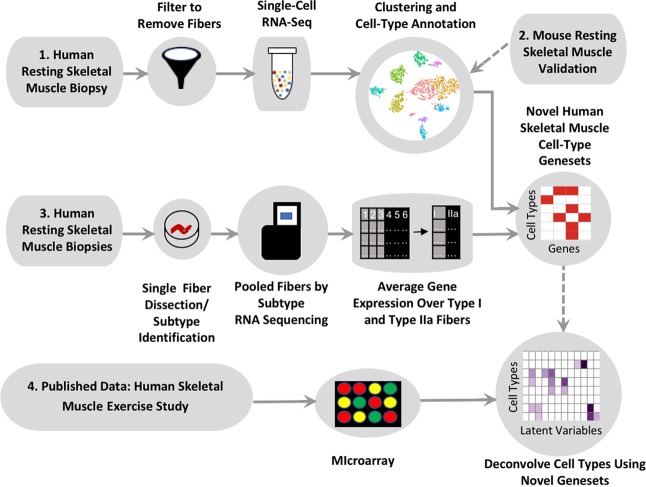
Schematic showing workflow of gene signature determination and skeletal muscle tissue deconvolution. Top (1): a skeletal muscle biopsy from the vastus lateralis obtained from a young healthy male was prepared by dissociation and filtering of skeletal muscle fibers. The cells were subjected to scRNA-seq using 10X Chromium. Normalization and clustering was performed by Seurat and clusters were manually identified as cell types. Top right (2): Confirmation of selected human skeletal muscle scRNA-seq cell types by mouse skeletal muscle scRNA-seq assay. Middle (3): skeletal muscle biopsies from the vastus lateralis of nine old healthy males were each used to isolate 96 individual muscle fibers. A small portion of each muscle fiber was clipped and subjected to SDS-PAGE to determine the MHC isoform. For each subject, all Type I fibers were pooled and all Type IIa fibers were pooled, for a total of 18 fiber-type specific samples which were then subjected to RNA-seq. After quality control and normalization, gene expression was averaged over all Type I samples and all Type IIa samples separately to obtain fiber-type specific gene signatures. Bottom (4): a previously published dataset^8^, consisting of microarray profiling of skeletal muscle biopsies from the vastus lateralis of 28 healthy young and old males and females was deconvolved using the previously determined mononuclear and multinucleated cell-type gene signatures. Biopsies were obtained before and 4 h after an acute resistance exercise bout at the onset and end of 12 weeks of resistance training (3 d/wk).

**Figure 2:**
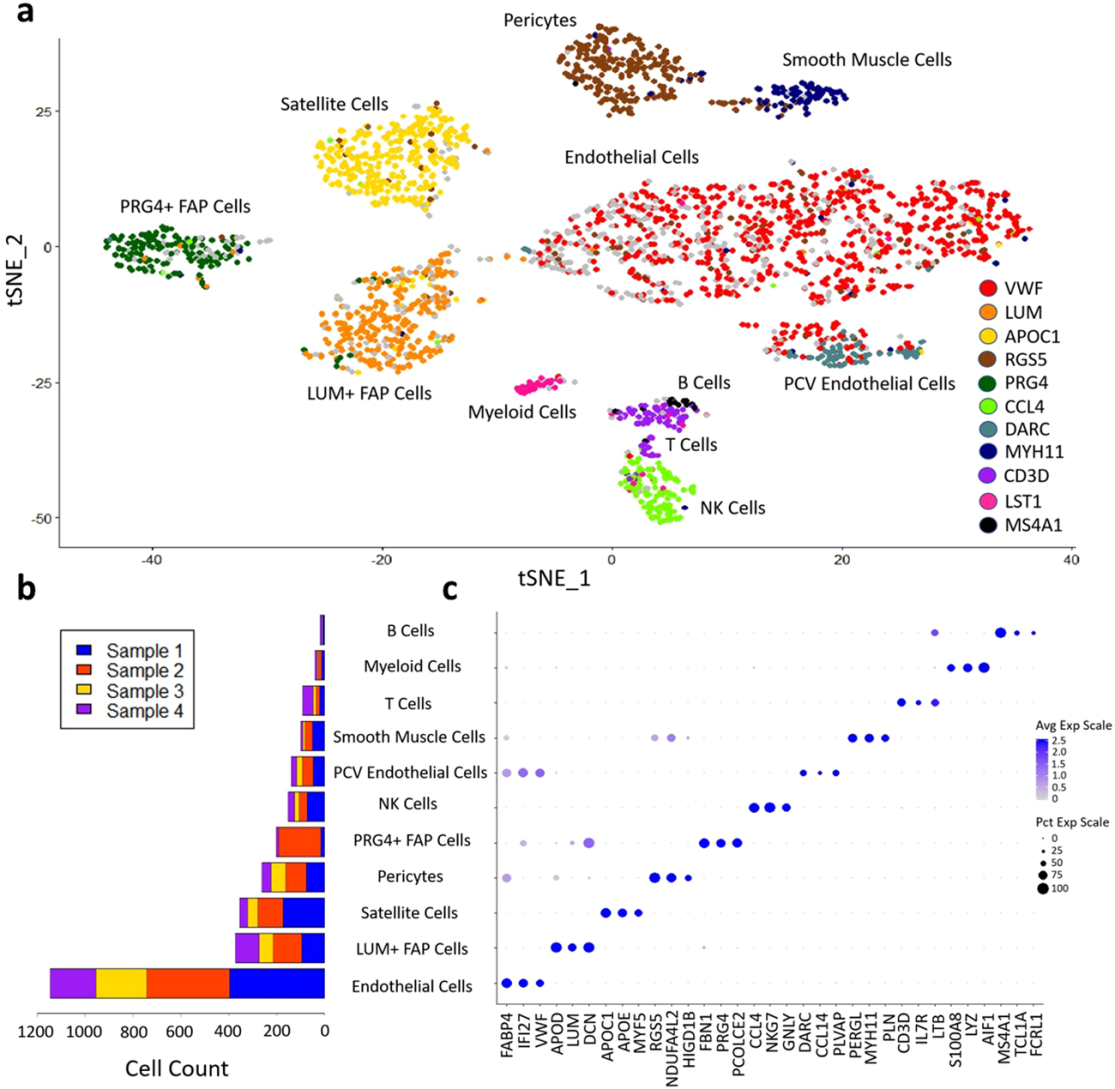
Single-cell RNA-seq cell-type analysis of human mononuclear muscle cells. (**a**) Cell-type labeled t-SNE plot of mononuclear cells from a combined set of four samples of a muscle biopsy. Cells are colored by their expression of top cell-type differentiating markers. Gray cells do not express any top cell-type differentiating markers, which may be due to transcript drop-out. (**b**) Bar graph of cell-type composition for each of the four muscle samples highlighting the heterogeneity of muscle cell-type composition. (**c**) Dot plot of gene expression of three top cell-type differentiating markers. Dot color reflects average gene expression and dot size represents percent of cells expressing the gene.

We assayed mononuclear cells in vastus lateralis skeletal muscle tissue by single-cell RNA-seq (scRNA-seq) to characterize the skeletal muscle cells that are not muscle fibers, their differences in gene expression and their heterogeneity (Fig. 1). We sequenced mononuclear cells isolated from four samples of a single muscle biopsy, which when computationally pooled together form a dataset comprising 108 million reads over 3,479 cells. The cells were clustered^28^ and then manually classified as known cell-type populations based upon their differential expression of known and novel marker genes (Methods, Supplementary Results). Figure 2a shows these distinct cell types in a t-SNE plot, where individual cells are colored by expression of a single top differentiating marker for each cell type. The groups could be distinguished using single marker genes, supporting the global marker-set based classification. Supplementary Table S1 lists the differentially expressed genes for each of the 11 mononuclear cell types described in this study.

Figure 2b details the frequency of each cell type in each of the samples to explore which cell type is most common and how cell-type composition varies from one sample to the next. As samples 1 and 2 both had roughly twice the amount of tissue and cell counts as samples 3 and 4, we expect approximately twice as many cells originating from samples 1 and 2 relative to samples 3 and 4 for cell types that are well-mixed and homogeneously distributed. Thus, cell types that do not conform to this ratio are more likely to be heterogeneous. Over 44% of cells were classified as endothelial cells, with a small minority representing a distinct gene expression profile reflecting endothelial cells restricted to post-capillary and small collecting venules. We refer to this population as post-capillary venule (PCV) endothelial cells. Both the main endothelial cell population and the PCV endothelial cells reflect the cell-type ratio expected of a homogenous population.

We also see large populations of satellite cells and pericytes. A large fraction of the differentially expressed genes in the satellite cell cluster are immediate/early genes (IEGs) which are activated in response to perturbation and in response to exercise (e.g. EGR1, JUNB, FOS, CYR61). Interestingly, a recent study found that some satellite cells respond to the dissociation protocol used in preparation for single-cell sequencing by overexpressing IEGs^29^. To investigate whether the satellite cells we found exhibited this behavior, we determined the percentage of transcripts from dissociation-affected genes in each single cell that we sequenced. We found that the percentages of dissociation-affected transcripts were generally within normal bounds, as defined by a threshold of 5.75% by van den Brink *et al*.^29^, although the satellite cell and myeloid cell populations generally expressed greater percentages of dissociation-affected genes than other cell types (Supplementary Fig. S1).

We identify two FAP cell clusters that both express the canonical FAP cell markers PDGFRA and CD34^30^. While CD34 is also expressed in vascular endothelial cells (Supplementary Fig. S2), among collagen-producing cells, both PDGFRA and CD34 expression is unique to FAPs^30^. PDGFRA and CD34 are not expressed in every cell in these clusters, which raises the possibility that mature fibroblasts could also be contained within each cluster; however, the gene expression of PDGFRA+/CD34+ cells is not significantly different compared to PDGFRA−/CD34− cells in these clusters, which suggests that either mature fibroblasts and their progenitors have very similar gene expression or the presence of mature fibroblasts within the clusters is very limited.

Despite both FAP clusters sharing important gene expression similarities, there are significant differences as well. The cells in the smaller FAP cluster express a series of genes that are commonly associated with synovial cell and chondrocyte expression. Genes that distinguish the two FAP cell clusters are shown in Supplementary Table S2. Based on the human and subsequent mouse muscle single cell RNA-seq analysis described below, we refer to the subtypes as fibrillin 1+ (FBN1+) and lumican+ (LUM+) FAP subtypes. The FAP cells show high heterogeneity across the samples. While the larger FAP cell cluster is consistently present within each sample, the FBN1+ FAP cells are enriched in one of the samples. This suggests that the FBN1+ FAPs are heterogeneously distributed. We also examined the collagen expression of both FAP cell types. Collagen types I, III and VI are expressed heavily in each cluster; however, there are three collagen types with distinguishing expression profiles (Supplementary Fig. S2): types IV, XIV and XV. The larger FAP cell cluster shows heightened expression of types IV and XV collagen, typically associated with expression in the basal lamina or endomysium of muscle^31^. Alternatively, the FBN1+ FAP cells show increased expression of type XIV collagen, which is typically associated with expression in muscle perimysium^32^.

A small population of smooth muscle cells are present within each sample, likely reflecting the vascular smooth muscle cells that compose the walls of blood vessels. The remaining cells in the samples were immune cells, with NK cells the most common, T cells the second most frequent and small numbers of B cells and myeloid cells also present.

We expand upon the distinctions in gene expression between cell types in Fig. 2c, where the average expression (dot pixel intensity) and cell expressing percentage (dot size) for three top marker genes for each cell type were compared. Expression levels across cell types are distinct from cell type to cell type for the marker genes. The greatest overlap is between the endothelial cells and their more distantly related PCV endothelial cells. We note that while the PCV endothelial cells do express the top marker genes from the main endothelial cell clusters, the top markers for PCV endothelial cells: DARC, PLVAP and CCL14, are not expressed in the remaining endothelial cell population. There is also some overlap between the two FAP cell clusters, as both cell types have overlapping structural gene expression. Both clusters express a high level of collagen types I and III, which are the predominant collagen fibrils in skeletal muscle interstitium. There is also considerable overlap in expression between the pericytes and the smooth muscle cell clusters in the expression of top pericyte markers. As expected, T and B cells share some overlap in expression as well.

**Figure 3:**
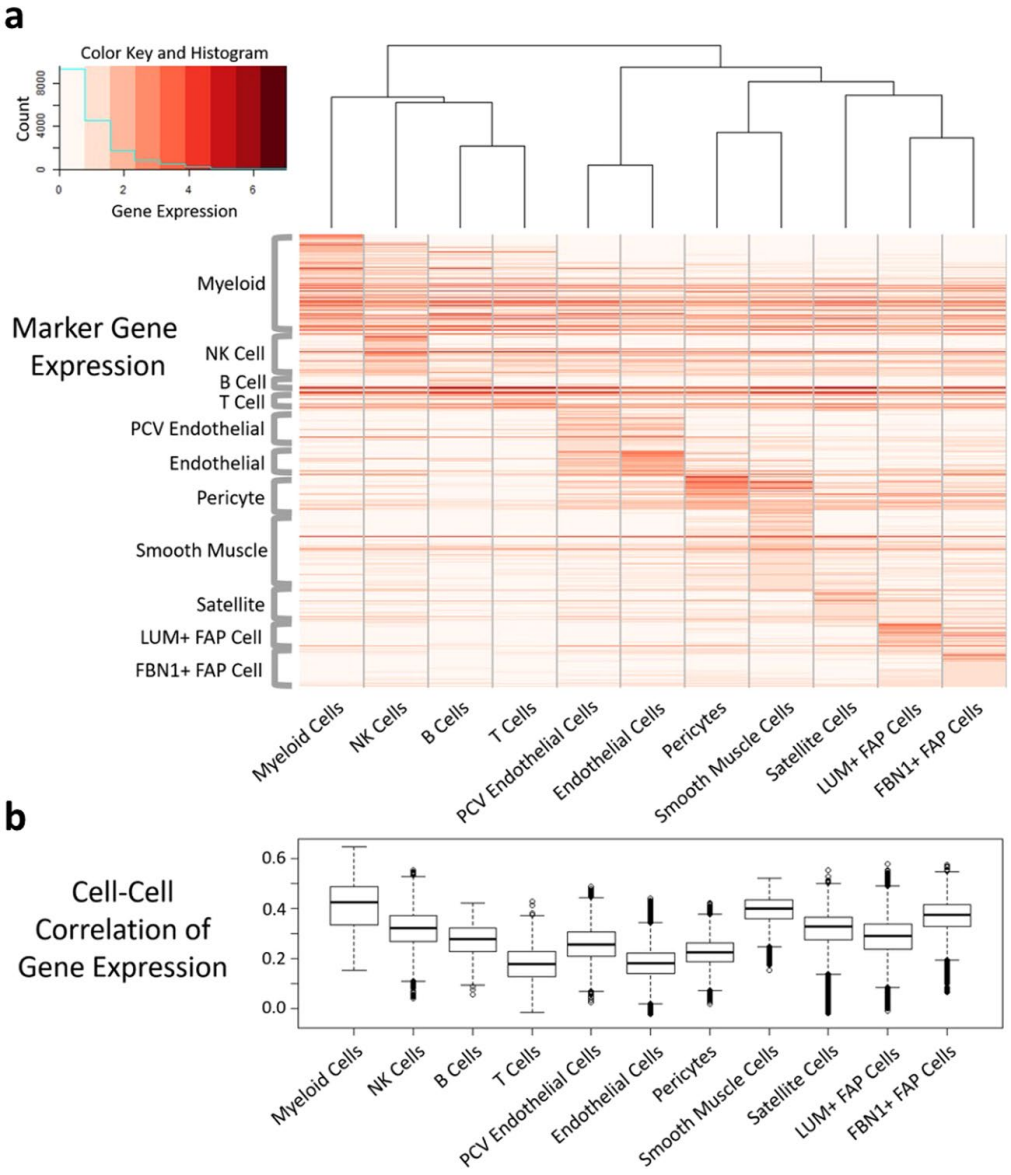
Human mononuclear muscle cell-type clustering analysis. (**a**) Heatmap and dendrogram of mononuclear cell types in muscle, clustered by their expression of top cell-type differentiating marker genes. (**b**) Box plot of cell-cell pearson correlation coefficients for each cell type. Higher values for a given cell type suggest greater homogeneity of gene expression between the cells of that cell type.

To investigate the relationships between the cell types further, we hierarchically clustered the cells via gene expression patterns. Figure 3a shows this analysis as part of a heatmap and dendrogram of clustered cell types on the x axis and the differentially expressed marker genes for each cell type on the y axis. Three main sets of cell types with similar expression profiles were identified. Not surprisingly, endothelial cells and PCV endothelial cells cluster together and the immune cell types - T cells, B cells, NK cells and myeloid cells - also cluster together, with T and B cells most similar in their gene expression. The remaining five cell types represent the final cluster, with the FAP cells clustering the most closely and smooth muscle cells and pericytes also highly clustered.

To better understand the homogeneity in gene expression within each cell type, a Pearson correlation coefficient was calculated for each pair of cells to determine the similarity in their gene expression profile, filtered on genes that varied significantly among cell types. Figure 3b shows a boxplot of the distribution in correlation coefficients for the pairs of genes in each cell type. The myeloid cells and the smooth muscle cells represent the most homogeneous populations with respect to their gene expression profiles (highest median correlation within cell type) and the endothelial cells are one of the more heterogeneous populations. The heterogeneity in the endothelial cells may be partially due to their large sample size, as they are by far the most common cell type in the dataset. On the other hand, despite their small size relative to that of the endothelial cell population, the T cell correlation value distribution is similar to the endothelial cells, which suggests that the T cells have truly diverse gene expression profiles, likely reflecting distinct T cell subtypes.

### Mouse single cell fap analysis

**Figure 4:**
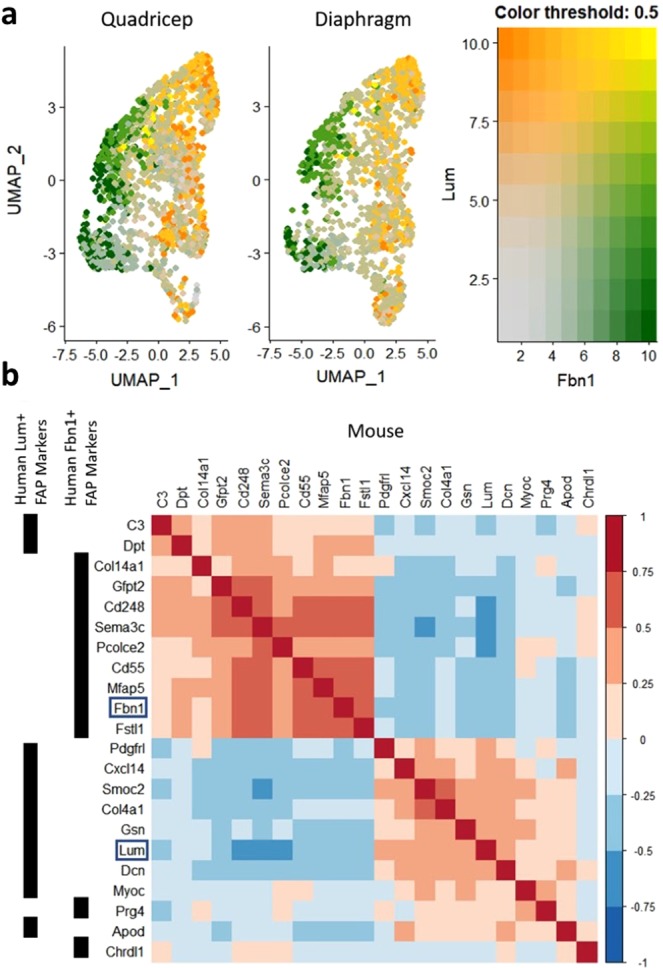
Single-cell RNA-seq cell-type analysis of mouse mononuclear muscle cells. (**a**) UMAP plot of FAP cells isolated from quadriceps and diaphragm, colored by their expression of FBN1 (green) and LUM (orange). (**b**) Correlation plot of the gene expression for 11 top markers for each human FAP cell subtype (FBN1+ and LUM+) over the mouse samples. Genes are arranged via hierarchical clustering with their human FAP subtype marker assignment labeled by the black bars on the left of the figure. The location of FBN1 and LUM are emphasized by boxes.

In order to validate the distinct FAP populations found in human muscle, we conducted a second single mononuclear cell sequencing study in two mouse muscle tissues: quadriceps and diaphragm, which were each obtained by pooling samples from 5 animals. We find that both FBN1+ and LUM+ FAP subtypes were identified in both mouse single-cell RNA-seq datasets (Fig. 4a). Because of the similarity of the FAP cells in both tissues, the datasets, containing a total of ~4000 cells, were merged for identification of FAP subtype specific markers. Distinguishing markers were highly correlated within the mouse FBN1+ and LUM+ FAP subtypes. Furthermore, the human and mouse genes that best distinguish FBN1+ and LUM+ FAP cells were largely consistent across species (Fig. 4b). Expression levels of the canonical FAP markers PDGFRA and CD34 are consistent among both FAP subtypes in both the human (Supplementary Figs. S2 and S3) and mouse (Supplementary Fig. S3) samples. Expression of the known FAP marker TEK is distinctive, however. While we do not see TEK expression in the human FAP cells, TEK is expressed in the mouse FAP cells, though limited to the FBN1+ FAP subtype. Confirming our gene expression correlation findings for top FAP subtype markers, clustering analysis of the mouse single cell data divides cells expressing human FBN1+ subtype markers from cells expressing human LUM+ subtype markers (Supplementary Fig. S3). Collagen expression in mouse FAP subtypes shares some similarity with human FAP subtypes. Collagen types I, III and VI are still broadly expressed across FAP subtypes and collagen types IV and XV are still predominantly expressed in LUM+ FAP cells; however, collagen type XIV is expressed in most FAP cells of both FBN1+ and LUM+ subtypes in mouse, whereas it is restricted to FBN1+ cells in human (Supplementary Fig. S4).

### Muscle fiber-type gene signatures

We also characterized the transcriptomes of the two major human skeletal muscle fiber types, Type I and Type IIa^33,34^, using single fiber segments. Classically, the differential expression of myosin heavy chain isoforms (MHC) delineates the identity of each fiber; Type I fibers express MYH7, whereas Type IIa fibers express MYH2^33,34^. We have previously reported transcriptome data from MHC I and MHC IIa muscle fibers in a small subset of young and old women, but the focus was on the exercise response in each fiber type^8^. Here, we sought to identify a coherent correlated fiber-type specific gene signature that would identify each fiber-type and provide the basis for fiber-type deconvolution of bulk skeletal muscle RNA-seq data.

Ninety-six individual muscle fibers (~5 mm segments) were manually isolated from each of nine vastus lateralis (VL) biopsies, obtained from elderly men (Fig. 1). The myosin heavy chain isoform of each muscle fiber was assessed by SDS-PAGE and the muscle fibers from each subtype (i.e. Type I or Type IIa) were pooled within each biopsy to generate fiber-type specific samples for RNA-seq analysis (Methods). After quality control and normalization, the transcriptomic profiles were averaged across the nine samples for each fiber-type. For each fiber-type, marker genes were identified by finding the twenty genes with the greatest fold change difference over the second fiber-type.

Among the marker genes, most had been previously reported as fiber-type specific (Table 1, Supplementary Table S4). Many marker genes encode isoforms of sarcomeric proteins that are the determinants of fiber type (e.g., myosin heavy chain, troponin, etc.); interestingly, both MYH2 and MYH1 were identified as Type IIa markers, although MYH1 is a canonical Type IIx marker. Since this was not detected at the protein level (via SDS-PAGE), the mRNA presence of MYH1 may indicate the inclusion of hybrid/transitional fibers in the pool of Type IIa muscle fibers. Additionally, some genes encode proteins involved in metabolism, with Type I metabolism-associated marker genes contributing to the higher oxidative capacity of Type I muscle fibers and Type IIa metabolism-associated marker genes contributing to the increased glycolytic capacity of Type IIa muscle fibers. Several marker genes are involved in calcium transport and the remaining five genes are prevalent in muscle but are of more general cellular function.

**Table 1:** Marker Genes for Type I and Type IIa muscle fibers.

Marker Genes	Type I (LFC)	Type IIa (LFC)
Sarcomeric	TPM3 (3.68) TNNC1 (3.35) TNNI1 (3.24) TNNT1 (3.47) MYH7 (3.19) MYL2 (3.29) MYL3 (3.50) MYL6B (2.71) ANKRD2 (1.30) MYOZ2 (3.18)	TPM1 (3.363) TNNC2 (2.86) TNNI2 (3.43) TNNT3 (3.81) MYH2 (3.67) MYH1 (1.72) MYL1 (1.59) MYLPF (3.14) MYBPC2 (3.11) ENO3 (1.67)
Calcium transport	ATP2A2 (3.12) CASQ2 (1.86) PLN (0.96)	ATP2A1 (2.65) SLN (1.56)
Metabolism	CD36 (1.14) *CYB5R1* (1.34) FABP3 (1.11) LDHB (2.59)	*ALDOA* (1.73) GAPDH (1.32) LDHA (1.57) *PKM* (1.31) PFKM (1.45) *PGM1* (1.03)
General	CA3 (1.18) *MYL12A* (1.32) PDLIM1 (2.59)	*DDIT4L* (0.97) *G0S2* (1.05)

Log_2_ fold-change vs. the opposite muscle fiber-type is in parentheses after each gene name. Italicized genes have not been previously identified as fiber-type specific, to the best of our knowledge.

**Figure 5:**
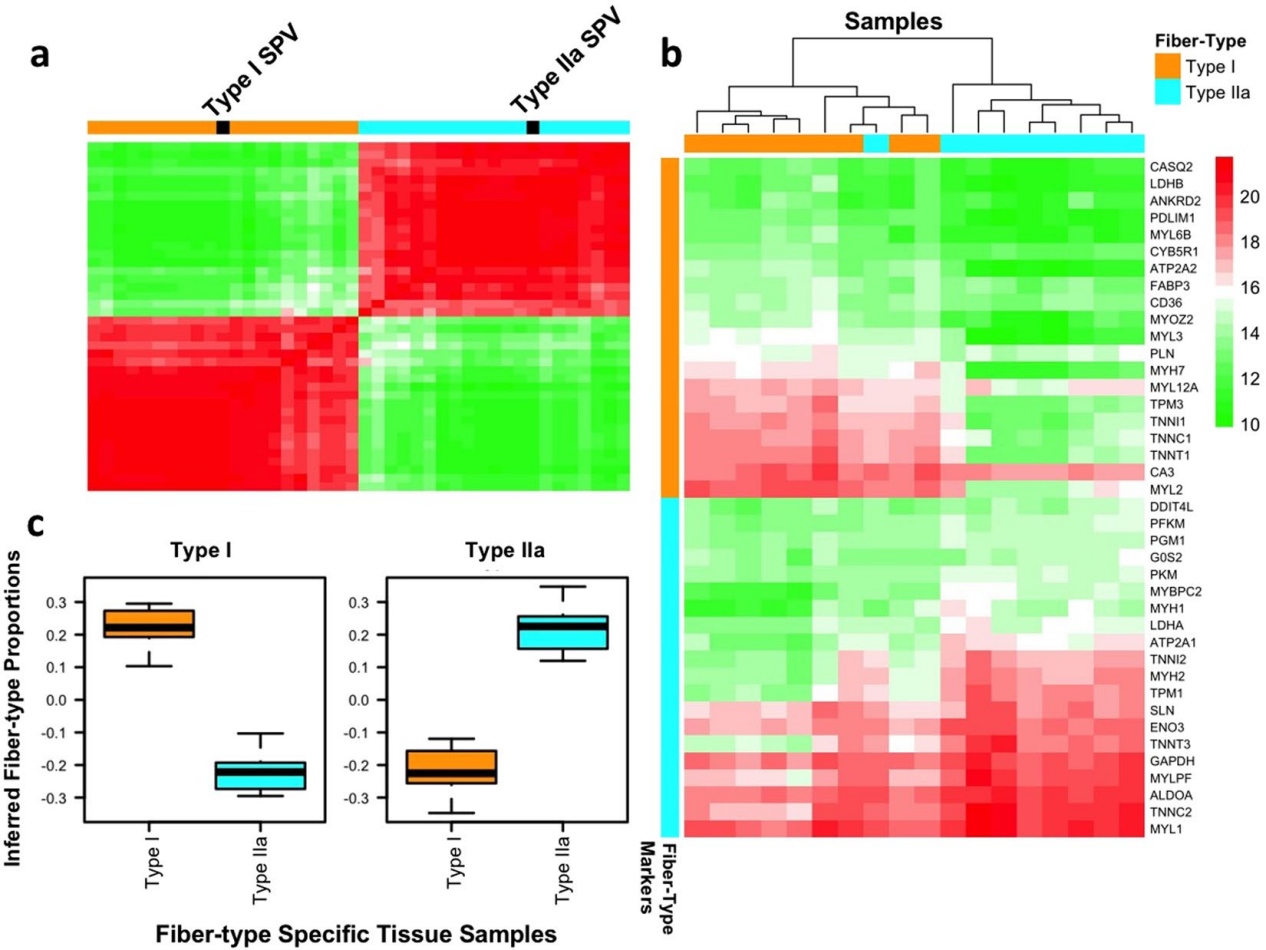
Fiber-type gene signatures and fiber-type specific tissue deconvolution. (**a**) Heatmap of gene expression for twenty markers per fiber-type over eighteen fiber-type specific tissue samples. Heatmap values are regularized-log transformed gene expression values. (**b**) Correlation heatmap for twenty gene markers per fiber-type. Estimated cell-type proportions (SPVs) for each fiber-type delineated in black; SPVs correlate with gene markers for each fiber-type. (**c**) Box plots showing estimated proportions of Type I fibers (left plot) and Type IIa fibers (right plot) within Type I specific tissue samples (orange boxes) and Type IIa specific tissue samples (blue boxes).

To investigate whether the fiber-type marker genes that we selected enable deconvolution of skeletal muscle tissue, the fiber-type specific tissue samples were analyzed using the CellCODE computational cell-type deconvolution framework^15^. As the proportions of fibers in the fiber-type specific tissue samples are known, the dataset is an optimal benchmark. High estimates of Type I proportion and low estimates of Type IIa proportion are expected in the Type I samples and the reverse is true for the Type IIa samples. Our analysis finds that the pairwise expression patterns between the marker genes for each fiber-type are highly correlated and cluster together in a block-like pattern (Fig. 5a), indicating that the expression levels of the fiber-type marker genes are similar within fiber-types and differ between fiber-types. The marker genes reliably distinguish the two groups of fiber-type samples, as the gene expression of the marker genes generally clusters by sample fiber-type (Fig. 5b). However, four samples (one Type IIa and three Type I) exhibited an expression pattern that fell between that of the two fiber-types. Finally, the inferred proportions of Type I fibers were high within fiber-type I samples and low in fiber-type IIa samples, while the reverse is true for Type IIa fibers, as is expected for fiber-type specific samples (Fig. 5c).

### Deconvolution of bulk transcriptomic profiles

**Figure 6:**
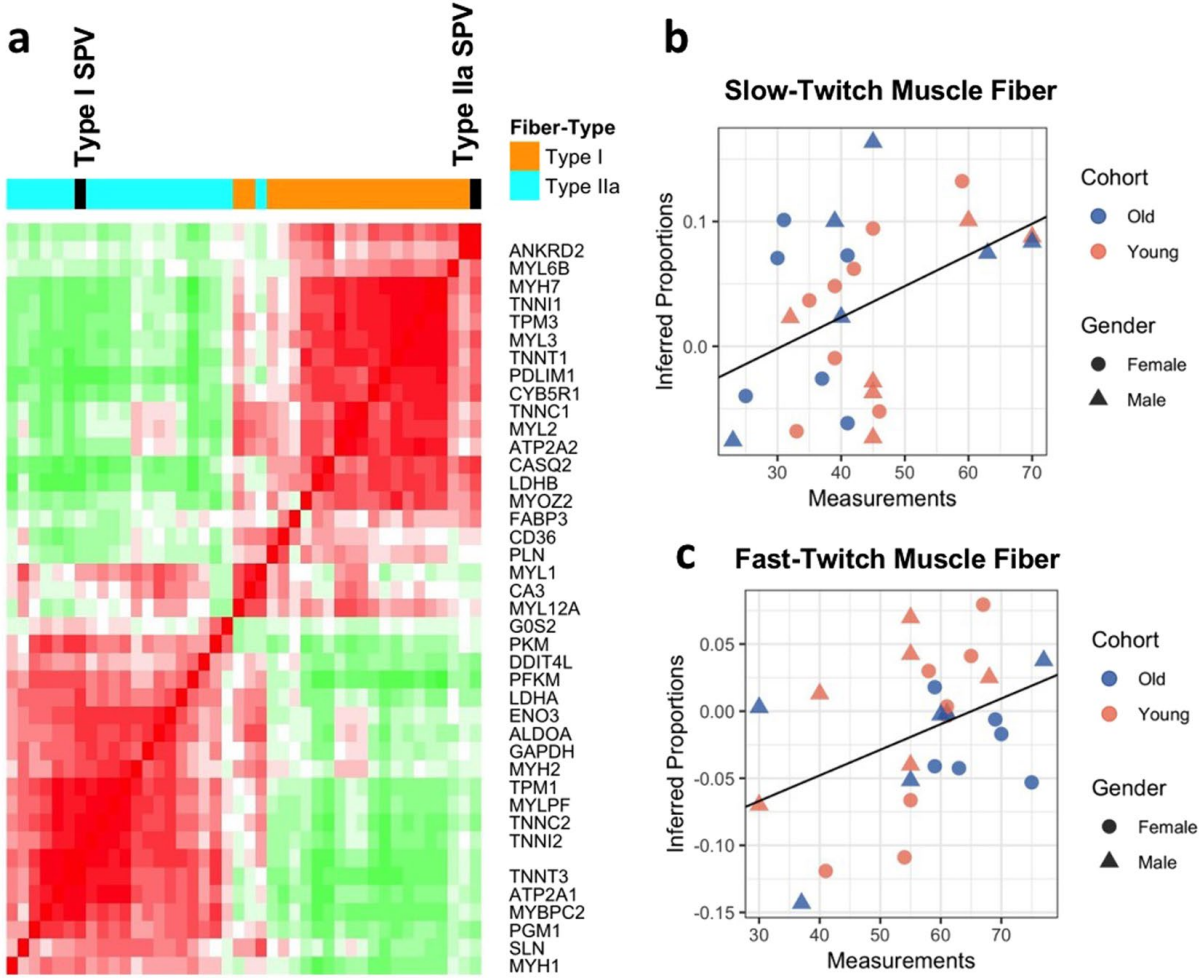
Fiber-type deconvolution of microarray dataset. (**a**) Correlation heatmap of twenty marker genes for each fiber-types. Estimated cell-type proportions (SPVs) for each fiber-type delineated in black; SPVs correlate with gene markers for each fiber-type. (**b**,**c**) Correlation of estimated Type I and Type IIa fiber-type proportions with biochemically measured slow-twitch and fast-twitch fiber-type proportions, respectively (slow-twitch: *p* = 0.02, fast-twitch: *p* = 0.03).

Genes often act in concert, such that the gene expression of multiple genes changes in a correlated manner between different samples. This correlated change may be due to a perturbation (e.g. exercise), differences between cohorts, or cell-type composition changes. Deconvolution algorithms track the correlated changes in gene expression to infer cell-type proportions. We benchmarked the ability to leverage the multinucleated and mononuclear gene signatures to deconvolve bulk skeletal muscle transcriptomic data. Using the new cell subtype skeletal muscle signatures we identified, we analyzed previously published skeletal muscle transcriptome dataset from a study on the effects of acute resistance exercise, resistance training, gender, and age on gene expression^8^. In our new analysis, the muscle cell-type signatures identified were used with CellCODE^15^ and the recently reported Pathway-Level Information ExtractoR (PLIER) method^16^ to infer changes in cell-type proportion. The 110 sample microarray dataset analyzed consists of vastus lateralis biopsies from young (24 ± 4 yr) and old (84 ± 3 yr) mixed gender cohorts, which were obtained at baseline and four hours post acute resistance exercise at the onset and end of 12 weeks of resistance training^8^. We first computationally estimated the proportions of Type I and Type IIa fibers within each sample using two panels of 20 fiber-type marker genes, identified above, for cell-type proportion deconvolution^15^. The vast majority of the pairwise correlations between the marker genes for each fiber-type are high and cluster together (creating a block-like pattern), indicating that the analysis tracks the proportions of the two major muscle fiber subtypes (Fig. 6a) and the derived fiber-type proportion estimates are reliable. To confirm the computational cell-type estimates, we compared the inferred fiber-type proportions within each sample with slow-twitch and fast-twitch fiber-type frequency estimates obtained from single fiber myosin heavy chain isoform determination. The significant correlation of the computational fiber type proportions with MHC isoform single fiber analysis (Fig. 6b,c, slow-twitch: r = 0.44, *p* = 0.02; fast-twitch: r = 0.42, *p* = 0.03) further supported that the computational deconvolution based on cell-type signatures provided reliable insight into cell-type proportion variation in this bulk transcriptome dataset.

**Figure 7:**
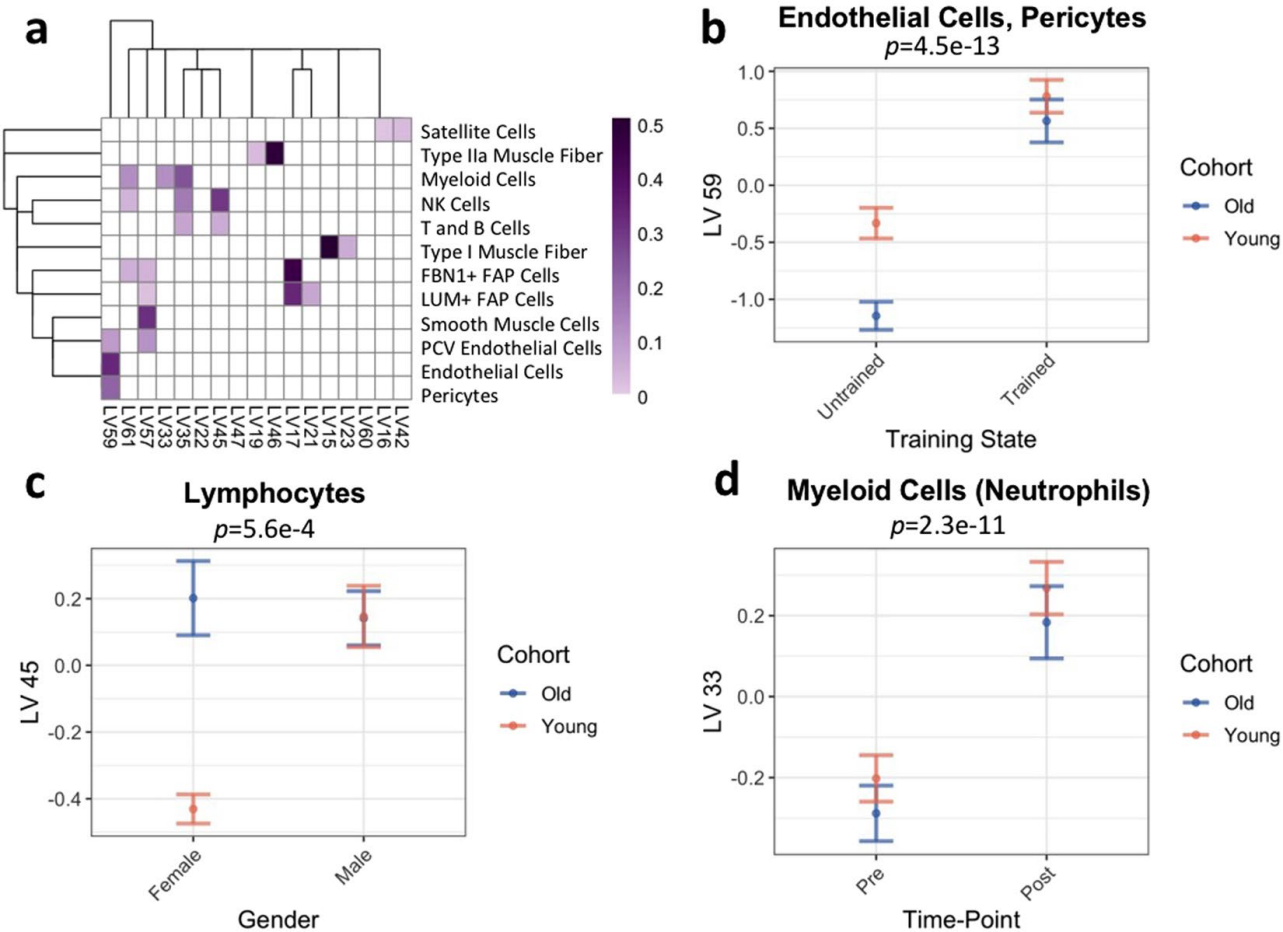
PLIER deconvolution of all skeletal muscle cell types. (**a**) Heatmap of association of cell types with LVs. The heatmap scale is arbitrary and should only be used to compare one association with another. (**b**) Difference in LV59 (endothelial cells/pericytes) between trained and untrained subjects. Points represent means of all trained and all untrained samples and error bars represent standard error. Both cohorts are depicted (orange for young and blue for old). (four-way ANOVA *p* = 4.5e–13) (**c**) Difference in LV45 (lymphocytes) between male and female subjects (four-way ANOVA *p* = 0.00056). Points represent means of all male and all female subjects and error bars represent standard error. Both cohorts are depicted (orange for young and blue for old). (**d**) Difference in LV33 (myeloid cells [neutrophils]) between pre- and post-acute exercise bout (four-way ANOVA *p* = 2.3e−11). Points represent means of all pre- and all 4 hour post-acute exercise samples and error bars represent standard error. Both cohorts are depicted (orange for young and blue for old).

Next, the transcriptomic profiles were analyzed for evidence of gene expression attributable to mononuclear cells resident in skeletal muscle, using PLIER^16^. Genes that are associated with a cell-type or biological pathway often change in a coordinated manner between samples, reflecting changes in cell-type composition or biological perturbation. PLIER extracts these correlated gene expression changes as a latent variable (LV). Simultaneously, PLIER maps each LV to prior knowledge (Fig. 7a, Supplementary Fig. S5). Prior knowledge is encapsulated as groups of genes (genesets) that have been reported to be associated with a biological pathway or cell type in public data repositories, such as KEGG or Reactome^35,36^. These genesets are thus based on multiple studies with different experimental techniques performed on a variety of cohorts. Since PLIER aligns multiple gene expression changes to genesets consisting of dozens of genes, it is agnostic to the modality used in finding the geneset and the cohorts involved. Additionally, as a data-driven method, PLIER only uses genesets that successfully map to correlated gene expression changes; genesets that do not show statistically significant correspondence to the dataset are eliminated. LVs that map to genesets for a given cell type can be used as a proxy for cell-type proportion (Fig. 7b–d, Supplementary Figs. S5, S6 and S7). As PLIER annotates LVs with known pathway and cell-type genesets, it is possible to identify existing biological knowledge that aligns with the skeletal muscle cell-type gene signatures.

The cell-type gene signatures, as determined using scRNA-seq and RNA-seq of single fiber segments, were used to generate genesets as input for PLIER, along with the default prior knowledge genesets in the PLIER package. We included genesets for twelve muscle cell types: Type I muscle fiber, Type IIa muscle fiber, endothelial cells, PCV endothelial cells, pericytes, LUM+ FAP cells, FBN1+ FAP cells, satellite cells, NK cells, T and B cells, smooth muscle cells, and myeloid cells (Supplementary Tables S3 and S4). In determining genesets, T cells and B cells were grouped together as their gene expression is highly similar and each population is small.

LVs that represent each of the twelve skeletal muscle cell types were found in the analysis of the bulk transcriptome dataset (Fig. 7a). Furthermore, LVs that associated with skeletal muscle cell types were annotated with biologically reasonable prior knowledge (Supplementary Fig. S5). For instance, of the three LVs that represented myeloid cell genesets, one LV associated with blood neutrophil signatures, one with blood mast/dendritic cell signatures, and one with blood monocyte/macrophage signatures as well as immune-related pathways.

Several of the mononuclear cell types exhibit exercise-, age-, and/or gender-related proportion changes. The endothelial cell/pericyte proportion (LV59) is higher in the trained group than in the untrained group (Fig. 7b, Supplementary Fig. S6). Additionally, in the untrained state, the endothelial cell proportion is higher in the young cohort than in the old cohort; after training, however, the endothelial cell proportion is consistent between the young and old cohorts. We also find that the lymphocyte proportion (LV45), consisting of NK cells, T cells, and B cells genesets, is lower in the young female group than in any other group (old female, young male, and old male) (Fig. 7c, Supplementary Fig. S7). This may be due to changes in immune profiles in pre-menopausal women. Finally, the neutrophil proportion (LV33), increased as a response to acute resistance exercise in all groups (trained and untrained, male and female) (Fig. 7d, Supplementary Fig. S7). These results demonstrate the generalizability of the cell type signatures we have identified and show how they can be used to provide cell type specific level insight from analysis of a bulk transcriptome muscle study.

## Discussion

We determined gene signatures for major mononuclear and multinucleated human skeletal muscle cell types, including a novel subtype of FAP that is identified in both human and mouse skeletal muscle samples. We applied these signatures to computationally identify changes in skeletal muscle cell-type proportions in response to acute exercise and resistance training using a bulk-transcriptome dataset. Comprehensive skeletal muscle cell-type signatures and the corresponding ability to reliably deconvolve skeletal muscle gene expression allows for the extension of bulk muscle transcriptome studies to cell-type resolution.

The identification of two distinct subpopulations of FAPs, distinguished in part by the expression of the FBN1 gene, has not been previously reported in human skeletal muscle. FAPs are non-myogenic mesenchymal stem cells that reside in muscle interstitium, are marked by expression of PDGFRA and CD34, and do not originate from bone marrow^11,13,22,37^. FAPs provide extracellular matrix (ECM) proteins for structural support to muscle and interact with myogenic stem cells to support myogenesis and muscle regeneration^11,13,38^. FAPs have also been implicated in myopathic fibrosis and fatty tissue replacement^13,22,37,39^. Additionally, Tie2+ FAPs in mice have been shown to exhibit chondrogenic/osteogenic potential, and are likely the major contributors to heterotopic ossification (HO) in skeletal muscle, a debilitating condition involving bone growth within skeletal muscle that results from trauma, central nervous system injuries, and genetic disorders^14,40–44^. Although TEK, the gene precursor of the Tie2 protein, is not highly expressed in either FAP subtype in human, the FBN1+ FAP subtype in mouse co-expresses the TEK gene. Thus, we speculate that the FBN1+ FAP subtype may be analogous to the Tie2+ FAP population in mice and may thus contribute to HO in humans as well. Additionally, the FBN1+ FAP cells specifically express elastin, MFAP5 and LOXL1 (Supplementary Table S1), three critical components of elastic fiber formation in the extracellular matrix, suggesting that these cells may play a role in elastic fiber assembly^45,46^.

While there is some degree of overlap, the transcriptional signatures of muscle-resident neutrophils, monocytes, NK, T cell and B cells, appear to be distinct from those of the corresponding circulating immune cells^25^ (Supplementary Table S5). Interestingly, there was some evidence of muscle gene expression (e.g. MYL6, TPM3) in the muscle-resident immune cells.

Using the cell-type signatures to computationally analyze cell-type proportion in a resistance exercise skeletal muscle transcriptome dataset, we identify an increase in the endothelial cell compartment with training, a decrease in lymphocyte proportion in young women, and an increase in neutrophil proportion with acute exercise. Studies that have examined changes in skeletal muscle capillarization (represented by endothelial cell compartment changes) with resistance exercise training are not entirely consistent, but overall capillarization appears to be influenced by age, training status, and training duration^47–49^. The 12 weeks of resistance training in the current study cohort may be too short to induce an increase in measurable capillary density; however, there may be an increase in gene expression within endothelial cells in the early stages of increasing capillary density, which is reflected in the change in the LV. Prior research has found that that sedentary elderly subjects have ~25% lower capillarization than sedentary young subjects, while elderly master athletes (i.e. lifelong exercisers) have similar capillarization to young athletes^50–52^; we find a similar pattern in endothelial cell compartment changes with training. Although there is little data regarding levels of immune cells in skeletal muscle during the menstrual-cycle, there are menstrual-cycle related differences in the proportions of circulating immune cell types^53^. Additionally, post-menopausal women have higher levels of CD8 T cells and NK cells and lower levels of CD4 T cells and B cells than do pre-menopausal women^54^, suggesting that LV45 specifically represents CD8 T cells and NK cells. Neutrophil infiltration of skeletal muscle in response to eccentrically-biased resistance exercise has been well-documented^55,56^. Previously, the increase in neutrophil proportion after intense, eccentric exercise has been shown to be attenuated with training^57^. However, we find that neutrophils increase with acute exercise to similar proportions in both the untrained and trained groups; this may be due to the lower intensity and the combination of concentric and eccentric contractions in the exercise regimen. The signatures we have identified will be similarly useful for applying similar computational approaches to infer cell subtype resolution insight from large scale bulk transcriptome muscle studies, such as the ongoing Molecular Transducers of Physical Activity (MoTrPAC) consortium which is studying the transcriptome response to exercise in muscle biopsies from thousands of subjects^58^.

The computationally inferred fiber-type proportions correlate significantly with experimental measurements but are not in complete correspondence. The difference between the estimates and the biochemically measured proportions may be due to the mismatch between the methods used: while the biochemical assay measures the relative proportions of MHC isoforms (MHC I vs. MHC IIa), the computational method relies on the abundance of multiple fiber-type marker genes. Additionally, the biochemical measurements categorize all fiber-types as either slow-twitch (MHC I, MHC I/IIa) or fast-twitch (MHC IIa, MHC IIx, MHC IIa/IIx), while the computational method is derived from Type I (MHC I) and Type IIa (MHC IIa) fibers only, so hybrid fibers may be categorized differently in the biochemical assay than in the computational estimates. This is especially relevant as the subjects in the microarray dataset are untrained and thus more likely to have hybrid fiber-types^6,7,59^. Finally, the biochemical assay measures the relative counts of fiber-types within the biopsy without considering fiber size, whereas computational estimates reflect total cellular mRNA content, comprising both fiber size and fiber count.

We determined gene signatures for a variety of cell types within human skeletal muscle, discovered a previously uncharacterized FAP subtype, and used the gene signatures to computationally infer cell-type proportions within bulk skeletal muscle tissue. Our study serves as a demonstration of computational analysis of bulk skeletal muscle transcriptomes at cell-type resolution, which will enable in-depth analysis of human muscle regulation in exercise, training, and disease. We note that our approach is limited in that a single biopsy is used to assay human skeletal muscle mononuclear cells, and that this biopsy is from a young male. It is possible that assaying more samples from different cohorts would yield a greater variety of cell types, especially rare ones. However, the cell-type gene signatures that we have identified encompass the major mononuclear cells found in muscle and, as we have shown, are extensible to deconvolution of skeletal muscle tissue from older males and females. Future studies of additional human subjects with a variety of ages, genders, and ethnicities, would be valuable.

## Methods

### Human participants and muscle sample collection

An outline of the subject participation is presented in Fig. 1. All procedures associated with the research were approved by the Institutional Review Board at Ball State University and performed in accordance with relevant guidelines and regulations. Subjects^8,52^ provided written informed consent prior to participation. Muscle biopsies were obtained from the vastus lateralis as we have previously described^8,52^.

### Human fiber-type specific samples

#### RNA-sequencing of slow- and fast-twitch muscle fiber samples

Eighteen fiber-type specific samples were generated as previously described^20^. Briefly, all samples were prepared according to the following steps: (1) manual muscle fiber isolation, (2) muscle fiber clipping to allow for fiber-type identification via SDS-PAGE^6^, (3) muscle fiber pooling of alike fiber-types, (4) RNA extraction (TRI Reagent, Molecular Research Center, Cincinnati, OH) and RNA quantification (Quant-iT RNA assay kit, Invitrogen, Carlsbad, CA). Cofactor Genomics (St Louis, MO) performed the library construction and RNA sequencing as previously described^20^. Briefly, the SMARTer® Ultra® Low Input RNA kit (Takara, Mountain View, CA) was used to generate cDNA from 9 ng of total RNA. Using the ThruPLEX® DNA-seq kit (Takara), 1–2 ng of double-stranded cDNA was end-repaired and A-tailed to prepare for adaptor ligation. Indexed adaptors were ligated to sample DNA, and the adaptor-ligated DNA library quality was assessed by measuring nanomolar concentration and the fragment size in base pairs. Sequencing was performed on the NextSeq 500 unit (Illumina, San Diego, CA). Samples were sequenced as single-end 75 bp, and an average of 41,810,217 reads were generated for each sample.

#### Analysis

Reads were subjected to quality control using FastQC^60^ and RNASeqMetrics (Picard)^61^, mapped using STAR^62^, and gene count summarized using featureCounts^63^. Counts were normalized using DESeq2 regularized-log transform function^64^ and averaged across all Type I samples and all Type IIa samples to obtain fiber-type specific transcriptomic profiles.

#### Deconvolution

Marker genes for each fiber-type were selected if the gene expression level in that fiber-type exceeded the gene expression level in the other fiber-type by a cutoff of 0.7 in log space (found previously to work well with normalized datasets)^15^. We used CellCODE’s default marker gene selection and cell-type proportion estimation algorithms; since there was no variable of interest in the dataset besides the fiber-type identity, we used the “raw” method instead of including a group interaction. Differential expression statistics for marker genes (e.g. LFC, p-values, etc.) were found using DESeq2^64^.

### Mononuclear cell scRNA-seq

#### Human mononuclear muscle cell isolation

One fresh skeletal muscle biopsy sample (~200 mg) was separated into four samples, immediately placed in ice-cold 1X PBS, and then minced in ice-cold 1X PBS.

Samples were thereafter incubated in a digestion solution mix (1.0 ml 5U collagenase and 1.0 ml 5U dispase II) at 37 **°**C until a homogenous solution was achieved (<1 hr), after which the enzymes were inactivated in cold 1X phosphate buffered saline (PBS) with 10% Fetal Bovine Serum (FBS). Each sample was then filtered through a 70 μm cell strainer, followed by centrifugation, 1X PBS-wash and additional centrifugation. The resultant pellet was re-suspended in cold 1X PBS and filtered through a 40 μm cell strainer followed by a final centrifugation step. The pellet was re-suspended in freezing media before being placed in a CoolCell FTS30 (BioCision, Mill Valley, CA) controlled-rate cell cryopreservation system. The dissociated cryopreserved cells were later thawed for library construction based on the 10X Genomics Demonstrated Protocol (Manual Part Number CG00039 Rev C).

#### Mouse mononuclear cell isolation

The mouse tissue collection protocols were approved by the Institutional Animal Care and Use Committees at the Boston University Medical Center (Boston, MA, USA) and the University of Texas Southwestern Medical Center (Dallas, TX, USA) and all experiments were performed in accordance with relevant named guidelines and regulations. Mouse quadriceps and diaphragm were collected from 10 mice (5 each) to minimize individual variability. Skeletal muscle single-cell suspension was prepared by collagenase/dispase digestion. Briefly, each tissue was minced in 2.5 ml digestion solution (1 U/ml collagenase B and 1 U/ml dispase II (Roche Diagnostics, Indianapolis, IN, USA) in PBS) and incubated at 37 °C for 1 hour. The reaction was terminated by adding 10 ml PBS containing 10% FBS. The mixture was then filtered through a 70-mm cell strainer and centrifuged at 250 g for 5 minutes. The pellet was collected and the supernatant was centrifuged again at 250 g for 5 minutes. The pellet was combined with the pellet from the first centrifugation, washed with PBS, and centrifuged at 670 g for 10 minutes. The pellet was re-suspended in 3 ml PBS and filtered through a 40-mm cell strainer. Cell suspension was layered on equal volume of the Lympholyte-M solution (Cedarlane, Burlington, NC, USA), and centrifuged at 2,095 g for 45 minutes. Cells at the interface were collected, centrifuged at 670 g for 10 minutes, and re-suspended in FACS staining buffer. Following this final centrifugation, cells from the five mouse diaphragms were collected into a single diaphragm sample and the cells from the five mouse quadriceps were collected into a single quadriceps sample. Viable skeletal muscle cells were then sorted by FACS based on FSC/SSC.

#### Single-cell RNA-sequencing

Cells were resuspended at final concentrations of ~200–1,000 cells/ul depending on the samples. Single-cell encapsulation with beads using 10× Genomics Chromium Single Cell 3′ kit was performed following the manufacturer instructions (User Guide Rev A, 10× Genomics, Pleasanton, CA, USA). Briefly, wells of a 10× microfluidic chip were loaded each with an individual sample. Single-cell gel beads in emulsion (GEMs) were generated and reverse-transcription was performed in the emulsions. Quality control and quantification of the amplified cDNA were assessed using standard protocols. Libraries were constructed and assessed according to the manufacturer instructions. Sequencing was performed using Illumina chemistry (Illumina, Inc., San Diego, CA, USA) and following 10× Genomics recommendations. Illumina sequencing output was generated for the samples as binary base call (BCL) files. Using the 10× Genomics cellranger pipeline^65^, the BCL files were demultiplexed and converted into FASTQ files.

#### Human mononuclear cell scRNA-seq analysis

The sequencing reads were aligned to the GRCh37 (hg19) human genome producing a raw counts matrix for each sample. In total, 108 million reads were matched to 3,479 cells across the four samples with a sequence saturation of approximately 80%. Following standard preliminary analysis to ensure sample quality (Supplementary Fig. S8)^28,65,66^, the samples were measured for the number of genes per cell in each sample, number of UMI per cell in each sample, and the percent of UMIs originating from mitochondrial genes per cell in each sample. For each metric, no significant differences between samples were found and most cells within each sample possessed a low percentage of mitochondria-originating UMIs, reflecting healthy cells. As the samples were of similarly good quality we pooled the four samples together into a single sample containing all 3,479 cells to strengthen the statistical power of our analysis.

We used Seurat^28^ to filter the cells in our combined sample to remove those with low gene counts, high mitochondrial RNA levels (>5%) and cells suspected of being doublets. Doublets were controlled by eliminating cells with high UMI counts in the Seurat 2.4 analysis suite and eliminating one cell cluster showing co-expression of FAP and endothelial markers that also showed ~ twice the average UMI levels/cell of those individual cell types. The final filtered count comprised 2,876 cells. Following normalization of UMI counts across cells and principal component analysis (PCA), cells were clustered over the first ten principal component dimensions, resulting in 11 clusters (Supplementary Fig. S9). Cell-type classification was completed using the canonical markers for individual muscle cell types and the known expression profiles for top differentially expressed genes in each cluster. Differentially expressed markers were derived by conducting a likelihood ratio test that determines a p-value based upon changes in mean expression and percentage of cells expressing genes^67^. Because clusters 0 and 1 have similar gene expression profiles, we consider them as one cell type, referred to as Endothelial Cells. Since cluster 7 has a much more distinct expression profile, we continue to refer to them as PCV endothelial cells. We also divided cluster 8 into separate T and B cell populations based upon their expression of CD3D vs MS4A1. We identified differentially expressed genes as putative surface markers if they were associated with the GO gene annotation for plasma membrane. A list of differentially expressed genes is found in Supplementary Table S1 and includes a column to denote whether the gene is a putative marker gene. A detailed description of differentially expressed markers and the cell-type classification process is available in the Supplementary Material.

#### Mouse mononuclear cell scRNA-seq analysis

The sequencing reads were aligned to the GRCm38 (mm10) mouse genome producing a raw counts matrix for each sample. In total, 725 million reads were matched to 7,658 cells across the two samples, again with a sequence saturation of approximately 80%. We used Seurat^28^ to filter cells with high mitochondrial content (>0.05%) and cells with low UMI counts or very high outlier UMI counts to exclude dead or dying cells and limit the risk of doublets. Following this filtering, the remaining 3,858 cells were normalized and clustered over the first 10 PCA components for each sample. Clusters comprised of FAP cells were isolated from each sample and subsequently merged into a single sample to increase classification power following the procedure of Seurat 3.1^68^.

### Microarray dataset for deconvolution

#### Microarray analysis

Skeletal muscle samples were prepared for microarray analysis as previously reported^8^. Briefly, total RNA was extracted from muscle samples homogenized in TRI-reagent (Molecular Research Center) according to manufacturer’s protocol. Each total RNA sample was purified using the RNeasy Micro Kit (Qiagen, Valencia, CA). The RNA quality was assessed using the Agilent 2100 Bioanalyzer (Agilent Technologies, Palo Alto, CA). RNA concentration was determined using the Quant-iT RNA assay kit (Invitrogen). RNA labeling, subsequent microarray hybridization, fluidics, and scanning for all samples were performed by Expression Analysis, Inc. (Durham, NC). Forty nanograms of total RNA from each muscle sample were labeled using the NuGen WT-Ovation RNA Amp v2 kit and protocol (NuGen, San Carlos, CA). Each sample was hybridized to an Affymetrix Human Genome U133 Plus 2.0 array (Affymetrix, Santa Clara, CA). Each microarray was washed and stained using an Affymetrix Fluidics Station 400 and scanned in an Affymetrix GeneChip Scanner 3000 7G scanner according to Affymetrix protocols.

#### Pre-processing

Microarray data was normalized using gcrma^69^. Probes with a maximum value below the threshold of 4.0 were removed. The probe with the highest median value over all samples was chosen as a representative for a single gene.

#### Deconvolution

To deconvolve fiber-types, we used CellCODE^15^ with default parameters. For each fiber-type, twenty marker genes were chosen from the fiber-type specific transcriptomes if the gene expression level exceeded the gene expression level in the second fiber-type by a threshold of 0.7 in log space. Only genes that were present in the microarray dataset were used for the selection of marker genes. During deconvolution, the group variables of age (young vs. old), gender (male vs. female), time-point (pre vs. post acute exercise), and training state (untrained vs. trained) were considered, to exclude a predefined percentage of potential marker genes that vary most across these groups.

We used PLIER^16^ for the deconvolution of all cell types (both mononuclear and multinucleated). Fifty marker genes were selected for each fiber-type using the method described above. Since many mononuclear cell types had overlapping marker genes, a different method was used to select marker genes for those populations. The top fifty differentially expressed genes for each cell type (determined by Seurat) were considered as the cell-type marker genes if the differential expression adjusted p-value was less than 0.1. Ribosomal proteins (genes beginning with “RPL” or “RPS”) were excluded as they were often found in the marker list due to subtle differences between clusters. T cells and B cells were grouped into one cluster to improve resolution. As the endothelial cell clusters were highly similar and cluster 1 had higher UMI counts than cluster 0 (Supplementary Results), we used cluster 1 only.

The PLIER^16^ default genesets matrix (blood cell markers and prior knowledge pathways) was augmented with the marker genes for both mononuclear and multinucleated human skeletal muscle cells, as described above. The parameter k, which controls the number of LVs generated, was set to 63, which is 150% of the number of statistically significant PCs (42 in this case), as is recommended^16^. Otherwise, default parameters were used for PLIER.

A PLIER-identified LV was chosen for analysis if the LV was associated with at least one pathway or the variance in the LV was greater than 0.001. The selected LVs were each evaluated using a four-way ANOVA with factors of gender (male and female), cohort (young and old), time-point (pre- and post-acute exercise), and training-state (untrained and trained) (n = 110 for each LV). Inspection of Q-Q plots of ANOVA residuals revealed approximate normality. The overall p-values and main effect p-values were adjusted over the selected LVs using the FDR^70^ procedure (once for the overall p-values and once for the main effect p-values).

#### Comparison of computational fiber-type estimates to MHC distribution

To evaluate the fiber-type deconvolution method used on the microarray study samples^8^, we compared the deconvolution data to the myosin heavy chain (MHC) distribution determined from those same subjects. MHC distribution was determined by single fiber MHC SDS-PAGE analysis as previously described^6^. Approximately 200 muscle fibers were isolated and analyzed from young women, old women, and old men (pre- and post-acute resistance exercise in the untrained state). One hundred muscle fibers were analyzed from the young men (pre-acute resistance exercise). Among all muscle fibers analyzed, a total of six MHC isoforms were detected (I, I/IIa, IIa, IIa/x, IIx, and I/IIa/IIx), with the slow MHC I and fast MHC IIa being the most common isoforms (75–80% of all muscle fibers). The hybrid muscle fibers MHC I/IIa and IIa/IIx collectively represented 20–25% of all muscle fibers analyzed. Since the fiber type signatures generated in CellCODE were based on RNA-Seq analysis from MHC I (Type I) and MHC IIa (Type IIa) muscle fibers, we reduced the 6 MHC isoforms detected via SDS-PAGE into two general classifications of “slow” and “fast”. MHC I and I/IIa muscle fibers were labeled “slow” and MHC IIa, IIax, IIx, and I/IIa/IIx were combined to form the “fast” classification. To compare the biochemically assayed fiber-type proportions with the Type I and Type IIa computationally inferred fiber-type proportions, the computationally inferred fiber-type proportions were averaged between pre- and post-acute exercise samples for old men, young women, and old women, whereas for the young men the computationally inferred fiber-type proportion for the pre-acute exercise samples was used. Correlation is measured using a two-sided Pearson correlation test (n = 26).

## Supplementary information

Supplementary Information.

Supplementary Table S1.

Supplementary Table S2.

Supplementary Table S3.

Supplementary Table S4.

Supplementary Table S5.

## Data Availability

Human single-cell RNA-seq data is available at the GEO repository under accession number GSE130646. Fiber-type specific RNA-seq data is available at the GEO repository under accession number GSE130977. Mouse single-cell RNA-seq data is available at the GEO repository under accession number GSE138707.
