# Supplementary material for: Single-cell transcriptional profiles in human skeletal muscle
Source: Sci Rep. 2020 Jan 14;10:229. doi: 10.1038/s41598-019-57110-6 (PMC6959232; doi:10.1038/s41598-019-57110-6)
Supplement: Supplementary file 1 — Supplementary Information. [file 41598_2019_57110_MOESM1_ESM.pdf]

Single-cell transcriptional profiles in human skeletal muscle

Rubenstein, Aliza B.*; Smith, Gregory R.*; Raue, Ulrika; Begue, Gwénaëlle; Minchev, Kiril; Ruf-Zamojski, Frederique; Nair, Venugopalan; Wang, Xingyu; Zhou, Lan; Zaslavsky, Elena; Trappe, Todd A.; Trappe, Scott; Sealfon, Stuart C.

Supplementary Results

Explanation of human mononuclear scRNA-seq cell-type identification

Following our single cell sequencing analysis pipeline discussed in the methods section, we divided our pooled muscle biopsy sample into 11 cell clusters (Supplementary Figure S9). Studying the gene expression of known cell-type markers and examining the genes that are differentially expressed within our cell types gave us the information necessary to assign cell-type labels to our clusters. In the ensuing sections, we discuss the distinct gene expression patterns for each of the cell types discovered in our sample.

Endothelial Cells

Three clusters in our sample can be classified as endothelial cells. Across clusters 0, 1 and 7, expression of the endothelial cell marker VWF[1] is pervasive, as well as other genes known to be highly expressed within endothelial cells such as aquaporin 1 (AQP1)[2], RNASE1[3], CD74[4], and integrin alpha 6 (ITGA6)[5]. In each case, these genes are barely expressed outside of the endothelial cell clusters. There is, however, some distinction to be had between clusters 0, 1 and 7. Clusters 0 and 1 are very similar in their genetic profiles. A log2 fold change (L2FC) of .52 is the largest difference in gene expression between the two clusters. Of the 50 genes that satisfy our minimum threshold to be considered differentially expressed, only three of them are more highly expressed in cluster 0 relative to cluster 1. This can be explained by examining the total UMI counts for the cells in cluster 0 compared to cluster 1, as seen in Supplementary Figure S10a. Indeed, the cells in cluster 1 on average have a higher UMI count than those in cluster 0; therefore, the endothelial cells in cluster 1 likely reflect those that were sequenced more deeply, or potentially they are larger cells with more RNA to be sequenced or include cells going

through different cell cycle stages, although no specific cell cycle markers are present. As can be seen in Supplementary Figure S10, sequencing depth can vary considerably between cells, even post-normalization. The major outlier among endothelial cell clusters is cluster 7, which contains 205 differentially expressed genes relative to clusters 0 and 1, 89 of which have a higher L2FC than the top marker of differential expression between clusters 0 and 1. The top differentiating marker for cluster 7 is duffy antigen/chemokine receptor (DARC) which has been shown to be exclusively expressed in post-capillary and small collecting venule endothelial cells and is completely absent from other potential endothelial cell populations[6]. This distinction led us to classify cluster 7 as post-capillary venule (PCV) endothelial cells.

One other interesting division of endothelial cell populations that is too slight to reflect a separate cluster is a group of cells in the main endothelial cell population that is lacking expression of AQP1 and ITGA6, two of the main endothelial cell marker genes. We can again look at differentially expressed markers between the AQP1+ and AQP1- populations. We find a set of genes that are overexpressed in AQP1- cells, topped by adenosylmethionine decarboxylase 1 (AMD1), which require further study. The divide between endothelial cells expressing AQP1 and AMD1 can be seen in Supplementary Figure S11a. This set of endothelial cells likely represents another location-dependent subset of endothelial cells as location is a major determinant of their function and gene expression.

LUM+ FAP and FBN1+ FAP Cells

Clusters 2 and 5 both express the two canonical markers for fibro/adipogenic progenitor (FAP) cells: platelet derived growth factor alpha (PDGFRA) and CD34 (Supplementary Figure S2a). PDGFRA expression is specific to FAPs[7] and, while CD34 is expressed in endothelial

cells, it is a distinguishing marker of FAPs from fibroblasts[8]. While both FAP clusters heavily express collagen types I, III and VI, collagen types IV, XIV and XV are differentially expressed by the two clusters (Supplementary Figure S2b) suggesting different localizations within muscle tissue.

Cluster 2 expresses a number of collagen-producing cell-specific markers, including apolipoprotein D (APOD) which is primarily found in fibroblasts near blood vessels[9]. Lumican (LUM) regulates collagen fibril assembly and is involved in fibril contractility and decorin (DCN) binds to type 1 collagen fibrils and is expressed in both cluster 2 and cluster 5[10]. Alcohol dehydrogenase 1B (ADH1B) expression is also highly specific to cluster 2 as well as myocilin (MYOC), which plays a role in cytoskeleton structural function and mutations in this gene can be a major cause of glaucoma[11]. The protein myocilin is normally expressed in corneal fibroblasts and is secreted in the aqueous humor of the eye.

Cluster 5 contains an interesting array of marker genes. The top marker gene for the cluster is fibrillin 1 (FBN1), which forms loose bundles of microfibrils within cartilage[12]. Other marker genes include PRG4, PCOLCE2, and MFAP5. Proteoglycan 4 (PRG4), also known as Lubricin, is primarily expressed within chondrocytes in the superficial and upper intermediate zones as well as synovial cells[13,14]. Procollagen c-endopeptidase enhancer 2 (PCOLCE2) is a cartilage marker as well, with increased expression in neocartilage[15]. Additionally, microfibrillar-associated protein 5 (MFAP5), also known as MAGP2, is often used a synovial cell marker[16]. This is surprising as our muscle biopsy was not located near a portion of muscle tissue that is believed to contain synovial cells or chondrocytes. We label these cells as FBN1+

FAP Cells, while we name Cluster 2 LUM+ FAP Cells to reflect the expression of two of their most distinguishing markers.

Satellite Cells

Cluster 3 expresses two canonical satellite cell markers: paired box protein 7 (PAX7)[17] and myogenic factor 5 (MYF5)[18]. Interestingly, the two genes with the strongest p-values for differential expression for cluster 3 have a less well-known relationship with satellite cells and are in fact two other apolipoproteins: apolipoprotein C1 (APOC1) and apolipoprotein E (APOE). Both lipoproteins were believed to be primarily expressed in the liver[19], although recent research has also targeted them for a role in late-onset Alzheimer's disease[20]. APOE has been shown to be expressed in skeletal muscle concentrated at neuromuscular junctions, which could be the source of its risk for neurodegenerative diseases such as Alzheimer's disease and Parkinson's disease[21].

Pericytes

The three canonical pericyte markers are CSPG4, also known as neuron-glial antigen 2 (NG2), beta-type platelet-derived growth factor (PDGFRB) and melanoma cell adhesion molecule (MCAM)[22], also known as CD146. While CSPG4 expression is very limited in our sample, each of these genes are most highly expressed in cluster 4. The marker with the strongest p-value is RGS5 whose expression is known to align strongly with PDGFRB and is a pericyte marker as well[23]. Neurogenic locus notch homolog protein 3 (NOTCH3) is also primarily expressed in cluster 4 whose expression in vascular tissue is restricted to mural cells, including pericytes[24].

NK Cells

Cluster 6 includes the expression of many NK cell-specific markers, such as natural killer cell granule protein 7 (NKG7)[25], granulysin (GNLY)[26] and granzyme A (GZMA)[27], all of which are only expressed in this cluster. Chemokine ligands 3,4,5 are all top markers for cluster 6, despite often also being associated with macrophages; however, research has shown all three to be expressed in NK cells as well[28]. NK cells can often be differentiated based on expression of CD16 (FCGR3A) and CD56 (NCAM1)[29]. Supplementary Figure S11b highlights the expression of these two marker genes in this cluster. While FCGR3A is expressed in cluster 6 cells, NCAM1 expression is very rare, which suggests cluster 6 consists of CD16+ CD56dim NK cells. Interestingly, both of these markers are expressed in other cell populations in our samples: FCGR3A is present in cluster 10 (myeloid cells, which is most probably due to the presence of CD16+ monocytes) and NCAM1 is lightly expressed in cluster 3 (satellite cells) highlighting the complex nature of marker genes in gene expression studies and the importance of context when using cell-type-classifying markers.

T & B Cells

Cluster 8 includes expression of lymphocyte-specific markers such as lymphotoxin beta (LTB)[30], interleukin-7 receptor-α (IL7R)[31] and L-selectin (SELL)[32]. CD52 is expressed in both monocytes and lymphocytes and we see it present in both clusters 8 and 10 as expected[33]. Because sequencing depth is limited in single cell data, we do not capture every canonical marker for T and B cells; however, we can still differentiate these two populations based on the markers that are present. Supplementary Figure S11c shows expression of CD3D, a T cell marker gene[34], and MS4A1, a B cell marker gene[35], within cluster 8. There is no overlap between cells expressing these two genes so while these cells cannot be divided by our clustering

algorithm because of their similarity in overall gene expression, we can still differentiate between these two cell types.

Smooth Muscle Cells

Cluster 9 exhibits expression of a number of top markers for both smooth muscle cells and myofibroblasts. Both alpha smooth muscle actin (ACTA2)[36] and transgelin (TAGLN)[37] are well known markers for myofibroblasts; however, they are both also expressed in smooth muscle cells. What does differentiate these cell types is expression of myosin, which is only seen in smooth muscle[38]. Smooth muscle myosin heavy chain (MYH11) is one of the top marker genes for cluster 9 and is expressed in a majority of cells in the cluster. Similar expression patterns are seen for smooth muscle myosin light kinase (MYLK) and myosin regulatory light polypeptide 9 (MYL9). Supplementary Figure S11d examines this comparison of expression between TAGLN and MYH11. All but two cells in the cluster express TAGLN, the myofibroblast marker, and a majority of cells express MYH11 as well. This does allow the possibility that some of these cells are myofibroblasts and they are clustered together with the smooth muscle cells.

Myeloid Cells

The smallest cluster in our sample is cluster 10 consisting of myeloid cells. This cluster likely includes multiple cell types of myeloid origin, including granulocytes, monocytes and mast cells, but because their individual populations are so small and their gene expression so similar, they cannot be divided nor treated as individual cell types. Top markers include S100 calcium binding proteins A8 and A9 which are secreted by active monocytes, granulocytes and neutrophils[39]. Lysozyme (LYZ), leukocyte-specific transcript 1 (LST1), allograft inflammatory

factor 1 (AIF1) and m-ficolin (FCN1) are all top markers for cluster 10 and are expressed specifically in myeloid-lineage cells[40–43].

Supplementary Methods

Comparison of blood- and muscle-resident immune cells

Fifty marker genes were found for each blood immune cell type in the IRIS dataset using the default CellCODE method, with a cutoff of 2.0 (as used previously)[44]. Then, for each muscle-resident immune cell type (neutrophils, monocytes, B cells, T cells, and NK cells), we normalized its transcriptomic profile in the context of the other blood immune cell types (e.g., for neutrophils, we normalized the muscle-resident myeloid cells relative to the transcriptome of blood monocytes, B cells, T cells, and NK cells) using quantile normalization. We then found fifty marker genes for the muscle-resident cell type relative to the other blood immune cell types using the default CellCODE method with a cutoff of 2. Finally, we compared the fifty marker genes for the blood immune cell type to the fifty marker genes for the muscle-resident immune cell type and found the marker genes that overlap.

Bibliography

1. Sadler, J. E. Biochemistry and genetics of von Willebrand factor. *Annu. Rev. Biochem.* **67,** 395–424 (1998).
2. Verkman, A. S. Aquaporin water channels and endothelial cell function. *J. Anat.* **200,** 617–627 (2002).
3. Landré, J. B. P. *et al.* Human endothelial cells selectively express large amounts of pancreatic-type ribonuclease (RNase 1). *J. Cell. Biochem.* **86,** 540–552 (2002).
4. Le Hiress, M. *et al.* Proinflammatory signature of the dysfunctional endothelium in pulmonary hypertension. role of the macrophage migration inhibitory factor/cd74 complex. *Am. J. Respir. Crit. Care Med.* **192,** 983–997 (2015).
5. Primo, L. *et al.* Increased expression of alpha6 integrin in endothelial cells unveils a proangiogenic role for basement membrane. *Cancer Res.* **70,** 5759–5769 (2010).
6. Thiriot, A. *et al.* Differential DARC/ACKR1 expression distinguishes venular from non-venular endothelial cells in murine tissues. *BMC Biol.* **15,** 45 (2017).
7. Low, M., Eisner, C. & Rossi, F. Fibro/adipogenic progenitors (faps): isolation by FACS and culture. *Methods Mol. Biol.* **1556,** 179–189 (2017).
8. Lin, G., Finger, E. & Gutierrez-Ramos, J. C. Expression of CD34 in endothelial cells, hematopoietic progenitors and nervous cells in fetal and adult mouse tissues. *Eur. J. Immunol.* **25,** 1508–1516 (1995).
9. Provost, P. R., Marcel, Y. L., Milne, R. W., Weech, P. K. & Rassart, E. Apolipoprotein D transcription occurs specifically in nonproliferating quiescent and senescent fibroblast cultures. *FEBS Lett.* **290,** 139–141 (1991).

10. Pulkkinen, L., Alitalo, T., Krusius, T. & Peltonen, L. Expression of decorin in human tissues and cell lines and defined chromosomal assignment of the gene locus (DCN). *Cytogenet. Cell Genet.* **60,** 107–111 (1992).

11. Wentz-Hunter, K., Shen, X. & Yue, B. Y. J. T. Distribution of myocilin, a glaucoma gene product, in human corneal fibroblasts. *Mol. Vis.* **9,** 308–314 (2003).

12. Keene, D. R. *et al.* Fibrillin-1 in human cartilage: developmental expression and formation of special banded fibers. *J. Histochem. Cytochem.* **45,** 1069–1082 (1997).

13. Alquraini, A. *et al.* The interaction of lubricin/proteoglycan 4 (PRG4) with toll-like receptors 2 and 4: an anti-inflammatory role of PRG4 in synovial fluid. *Arthritis Res. Ther.* **17,** 353 (2015).

14. Jay, G. D. & Waller, K. A. The biology of lubricin: near frictionless joint motion. *Matrix Biol.* **39,** 17–24 (2014).

15. Wilson, R. *et al.* Comprehensive profiling of cartilage extracellular matrix formation and maturation using sequential extraction and label-free quantitative proteomics. *Mol. Cell. Proteomics* **9,** 1296–1313 (2010).

16. Rapko, S. *et al.* Identification of the chondrocyte lineage using microfibril-associated glycoprotein-2, a novel marker that distinguishes chondrocytes from synovial cells. *Tissue Eng. Part C Methods* **16,** 1367–1375 (2010).

17. von Maltzahn, J., Jones, A. E., Parks, R. J. & Rudnicki, M. A. Pax7 is critical for the normal function of satellite cells in adult skeletal muscle. *Proc Natl Acad Sci USA* **110,** 16474–16479 (2013).

18. Beauchamp, J. R. *et al.* Expression of CD34 and Myf5 defines the majority of quiescent

adult skeletal muscle satellite cells. *J. Cell Biol.* **151,** 1221–1234 (2000).

19. Cudaback, E. *et al.* Apolipoprotein C-I is an APOE genotype-dependent suppressor of glial activation. *J. Neuroinflammation* **9,** 192 (2012).

20. Yamazaki, Y., Painter, M. M., Bu, G. & Kanekiyo, T. Apolipoprotein E as a therapeutic target in alzheimer's disease: A review of basic research and clinical evidence. *CNS Drugs* **30,** 773–789 (2016).

21. Akaaboune, M., Villanova, M., Festoff, B. W., Verdière-Sahuqué, M. & Hantaï, D. Apolipoprotein E expression at neuromuscular junctions in mouse, rat and human skeletal muscle. *FEBS Lett.* **351,** 246–248 (1994).

22. Birbrair, A. *et al.* Role of pericytes in skeletal muscle regeneration and fat accumulation. *Stem Cells Dev.* **22,** 2298–2314 (2013).

23. Cho, H., Kozasa, T., Bondjers, C., Betsholtz, C. & Kehrl, J. H. Pericyte-specific expression of Rgs5: implications for PDGF and EDG receptor signaling during vascular maturation. *FASEB J.* **17,** 440–442 (2003).

24. Kofler, N. M., Cuervo, H., Uh, M. K., Murtomäki, A. & Kitajewski, J. Combined deficiency of Notch1 and Notch3 causes pericyte dysfunction, models CADASIL, and results in arteriovenous malformations. *Sci. Rep.* **5,** 16449 (2015).

25. Turman, M. A., Yabe, T., McSherry, C., Bach, F. H. & Houchins, J. P. Characterization of a novel gene (NKG7) on human chromosome 19 that is expressed in natural killer cells and T cells. *Hum. Immunol.* **36,** 34–40 (1993).

26. Latinovic-Golic, S. *et al.* Expression, processing and transcriptional regulation of granulysin in short-term activated human lymphocytes. *BMC Immunol.* **8,** 9 (2007).

27. Grossman, W. J. *et al.* Differential expression of granzymes A and B in human cytotoxic lymphocyte subsets and T regulatory cells. *Blood* **104,** 2840–2848 (2004).

28. Robertson, M. J. Role of chemokines in the biology of natural killer cells. *J. Leukoc. Biol.* **71,** 173–183 (2002).

29. Poli, A. *et al.* CD56bright natural killer (NK) cells: an important NK cell subset. *Immunology* **126,** 458–465 (2009).

30. Wicks, K. & Knight, J. C. Transcriptional repression and DNA looping associated with a novel regulatory element in the final exon of the lymphotoxin-β gene. *Genes Immun.* **12,** 126–135 (2011).

31. Passtoors, W. M. *et al.* IL7R gene expression network associates with human healthy ageing. *Immun. Ageing* **12,** 21 (2015).

32. Kohn, L. A. *et al.* Lymphoid priming in human bone marrow begins before expression of CD10 with upregulation of L-selectin. *Nat. Immunol.* **13,** 963–971 (2012).

33. Stevenson, E. V., McGee, J., Alexander, J. S. & Minagar, A. *Multiple Sclerosis: A Mechanistic View*. 13–21 (Academic Press, 2016).

34. Ohno, H. *et al.* Developmental and functional impairment of T cells in mice lacking CD3 zeta chains. *EMBO J.* **12,** 4357–4366 (1993).

35. Tedder, T. F. & Engel, P. CD20: a regulator of cell-cycle progression of B lymphocytes. *Immunol. Today* **15,** 450–454 (1994).

36. Nagamoto, T., Eguchi, G. & Beebe, D. C. Alpha-smooth muscle actin expression in cultured lens epithelial cells. *Invest. Ophthalmol. Vis. Sci.* **41,** 1122–1129 (2000).

37. Sheffer, Y. *et al.* Inhibition of fibroblast to myofibroblast transition by halofuginone

contributes to the chemotherapy-mediated antitumoral effect. *Mol. Cancer Ther.* **6,** 570–577 (2007).

38. Latif, N., Sarathchandra, P., Chester, A. H. & Yacoub, M. H. Expression of smooth muscle cell markers and co-activators in calcified aortic valves. *Eur. Heart J.* **36,** 1335–1345 (2015).

39. Kato, J. & Svensson, C. I. Role of extracellular damage-associated molecular pattern molecules (DAMPs) as mediators of persistent pain. *Prog. Mol. Biol. Transl. Sci.* **131,** 251–279 (2015).

40. Keshav, S., Chung, P., Milon, G. & Gordon, S. Lysozyme is an inducible marker of macrophage activation in murine tissues as demonstrated by in situ hybridization. *J. Exp. Med.* **174,** 1049–1058 (1991).

41. Fukui, M. *et al.* The serum concentration of allograft inflammatory factor-1 is correlated with metabolic parameters in healthy subjects. *Metab. Clin. Exp.* **61,** 1021–1025 (2012).

42. Draber, P. *et al.* LST1/A is a myeloid leukocyte-specific transmembrane adaptor protein recruiting protein tyrosine phosphatases SHP-1 and SHP-2 to the plasma membrane. *J. Biol. Chem.* **287,** 22812–22821 (2012).

43. Teh, C., Le, Y., Lee, S. H. & Lu, J. M-ficolin is expressed on monocytes and is a lectin binding to N-acetyl-D-glucosamine and mediates monocyte adhesion and phagocytosis of Escherichia coli. *Immunology* **101,** 225–232 (2000).

44. Chikina, M., Zaslavsky, E. & Sealfon, S. C. CellCODE: a robust latent variable approach to differential expression analysis for heterogeneous cell populations. *Bioinformatics* **31,** 1584–1591 (2015).

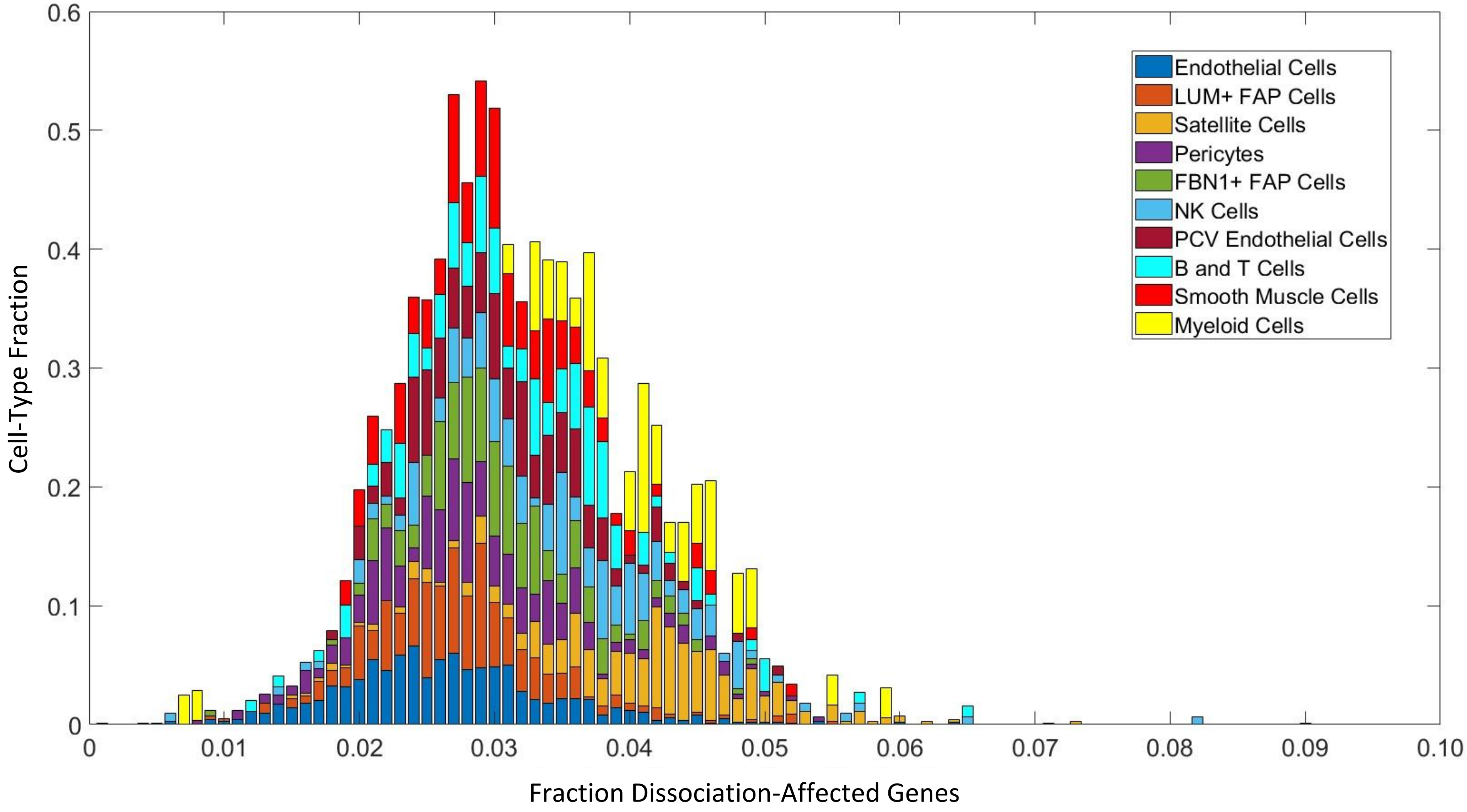

Figure S1: Cell type-specific expression of dissociation-affected genes. Stacked histograms of the fraction of cells for each cell type that contain a given fraction of dissociation-affected genes. Satellite cells (gold) and myeloid cells (yellow) possess the highest-skewing distribution of dissociation-affected gene fractions.

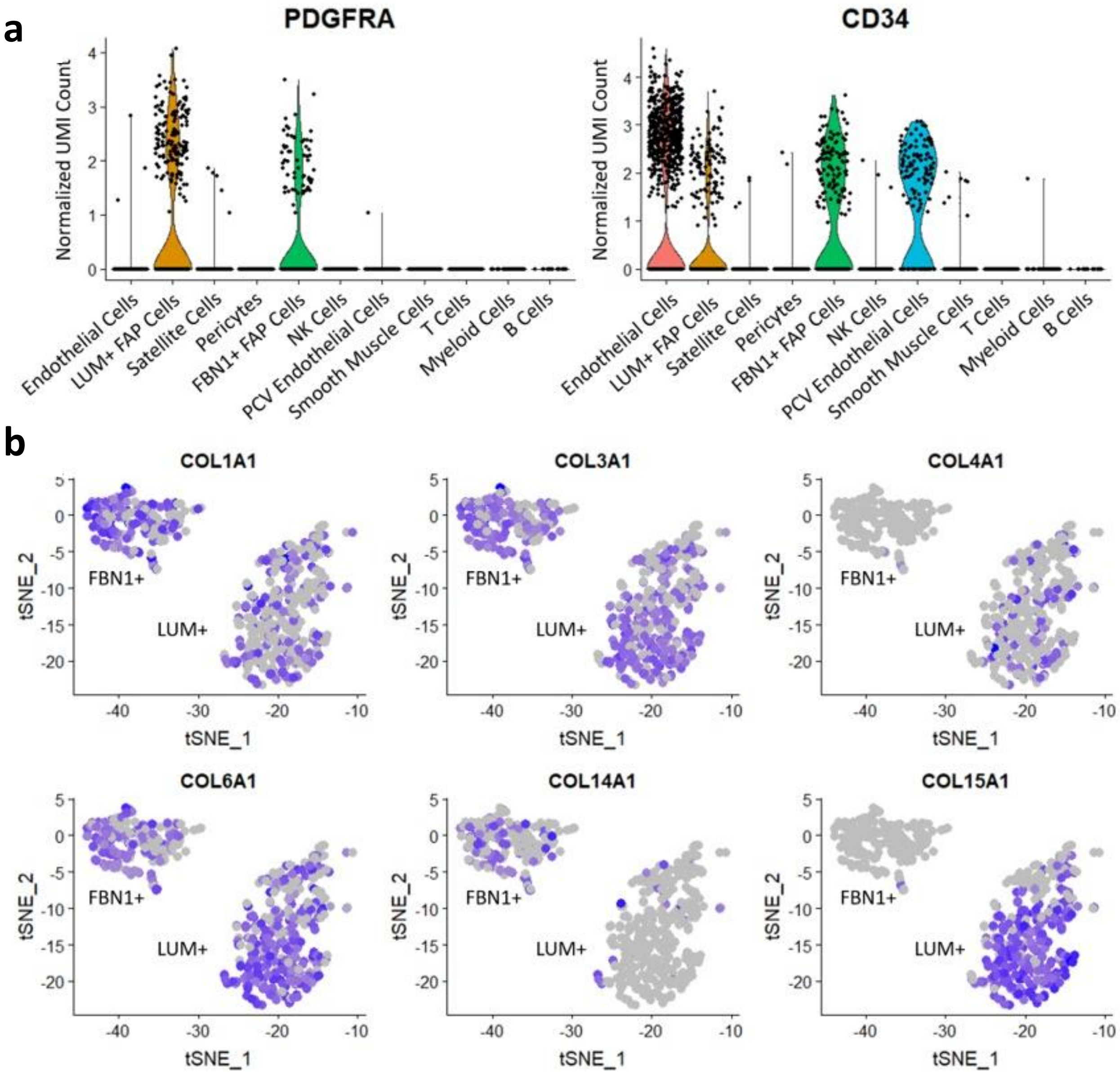

Figure S2: Gene expression of canonical FAP markers and collagen subtypes in human single-cell RNA sequenced samples. (A) Violin plot of gene expression for FAP canonical markers in each cell type. (B) Plots of cells in the FAP clusters, colored by expression of each collagen gene.

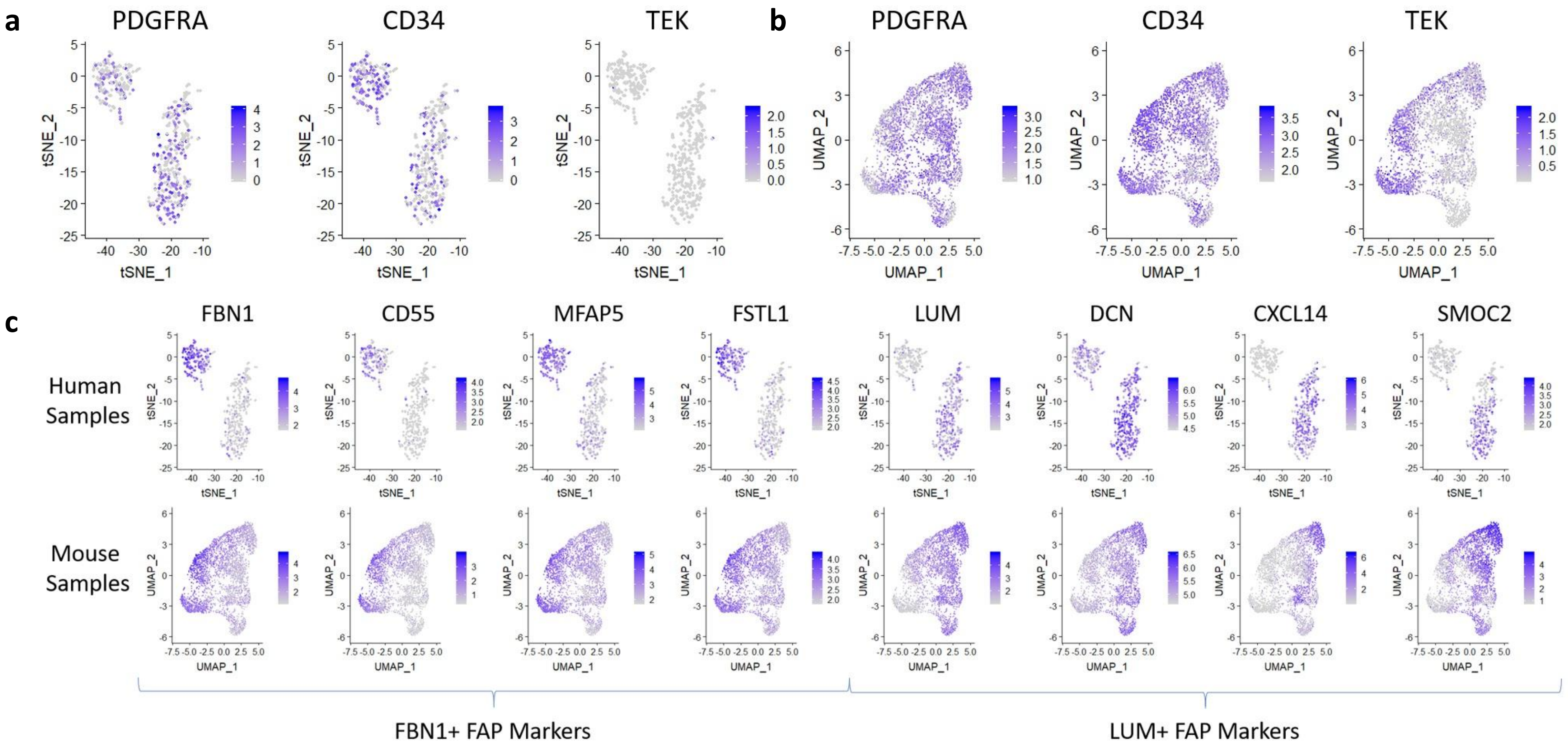

Figure S3: Gene expression of canonical FAP markers and FAP-subtype differentiating markers in human and mouse single-cell RNA sequenced samples. (A) Plots of cells in human FAP clusters, colored by expression of canonical FAP markers. (B) Plots of cells in mouse FAP clusters, colored by expression of canonical FAP markers. (C) Plots of cells in human (first row) and mouse (second row) FAP clusters, colored by expression of FAP-subtype differentiating markers. The four genes on the left are FBN1+ FAP markers and the four genes on the right are LUM+ FAP markers.

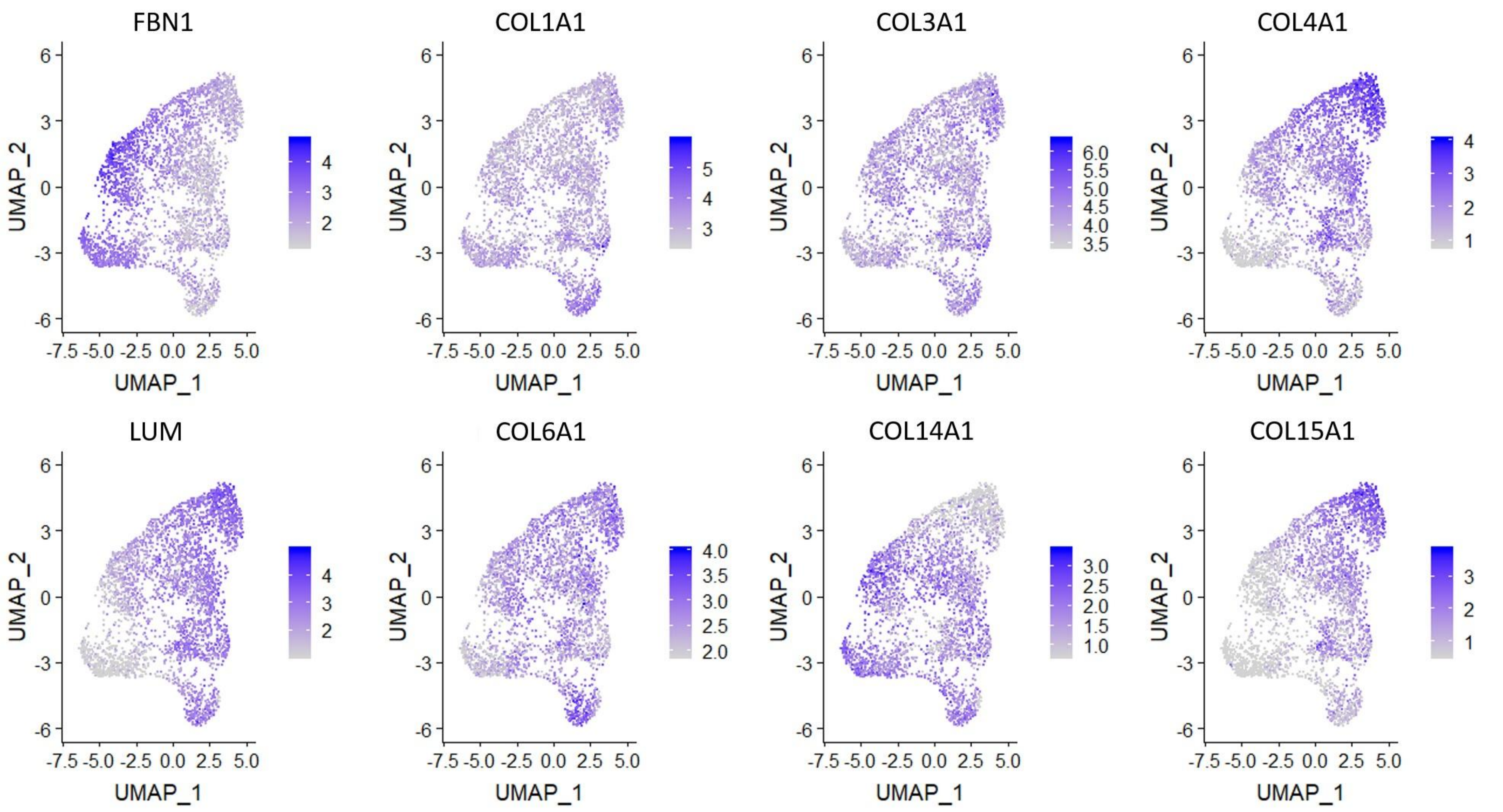

Figure S4: Plots of cells in the mouse FAP clusters, colored by expression of each collagen gene. FBN1 and LUM are included for the sake of labeling of FAP-type populations.

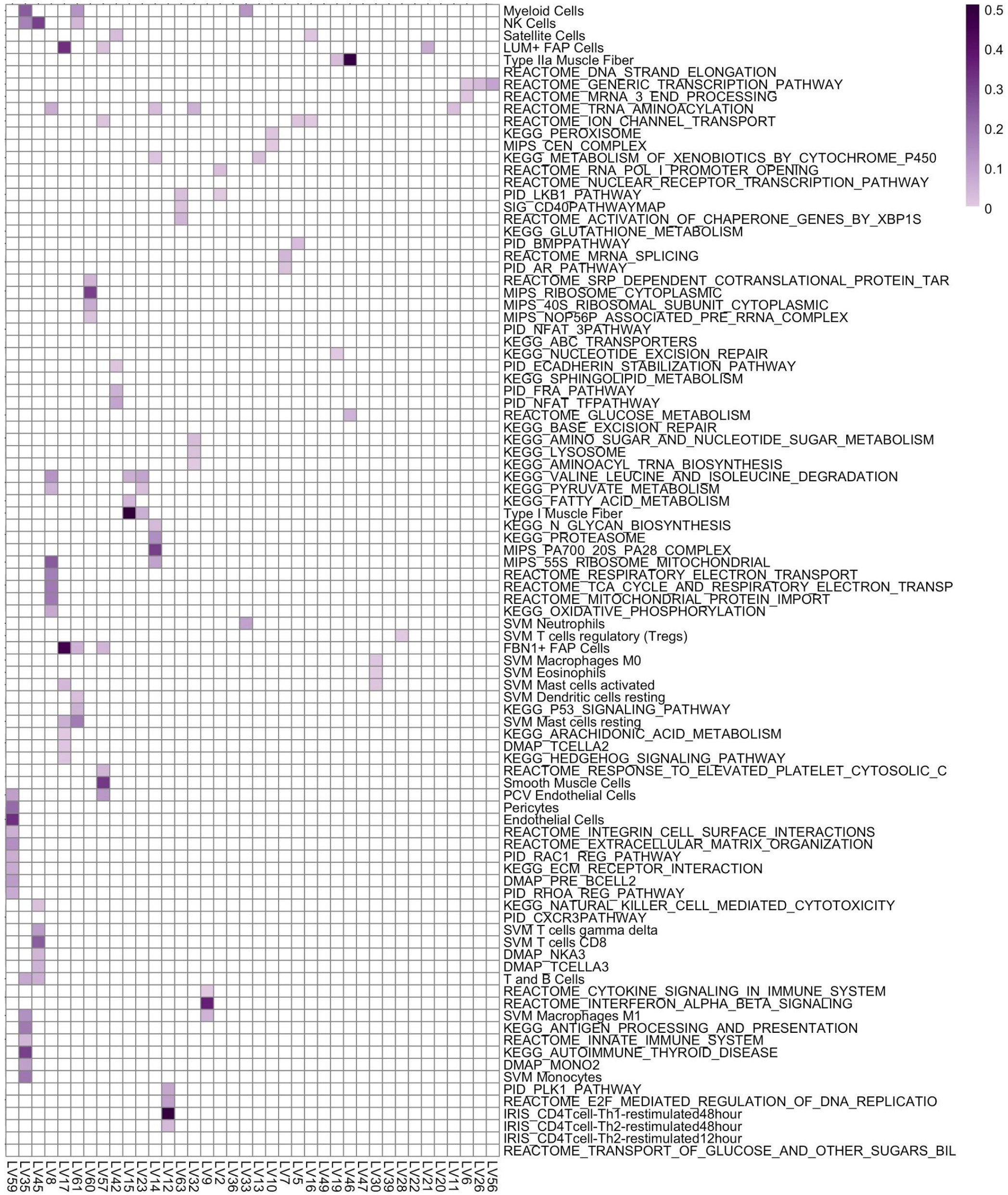

Figure S5: Heatmap of association of LVs with all genesets. The heatmap scale is arbitrary and should only be used to compare one association with another. Columns represent LVs and rows represent genesets.

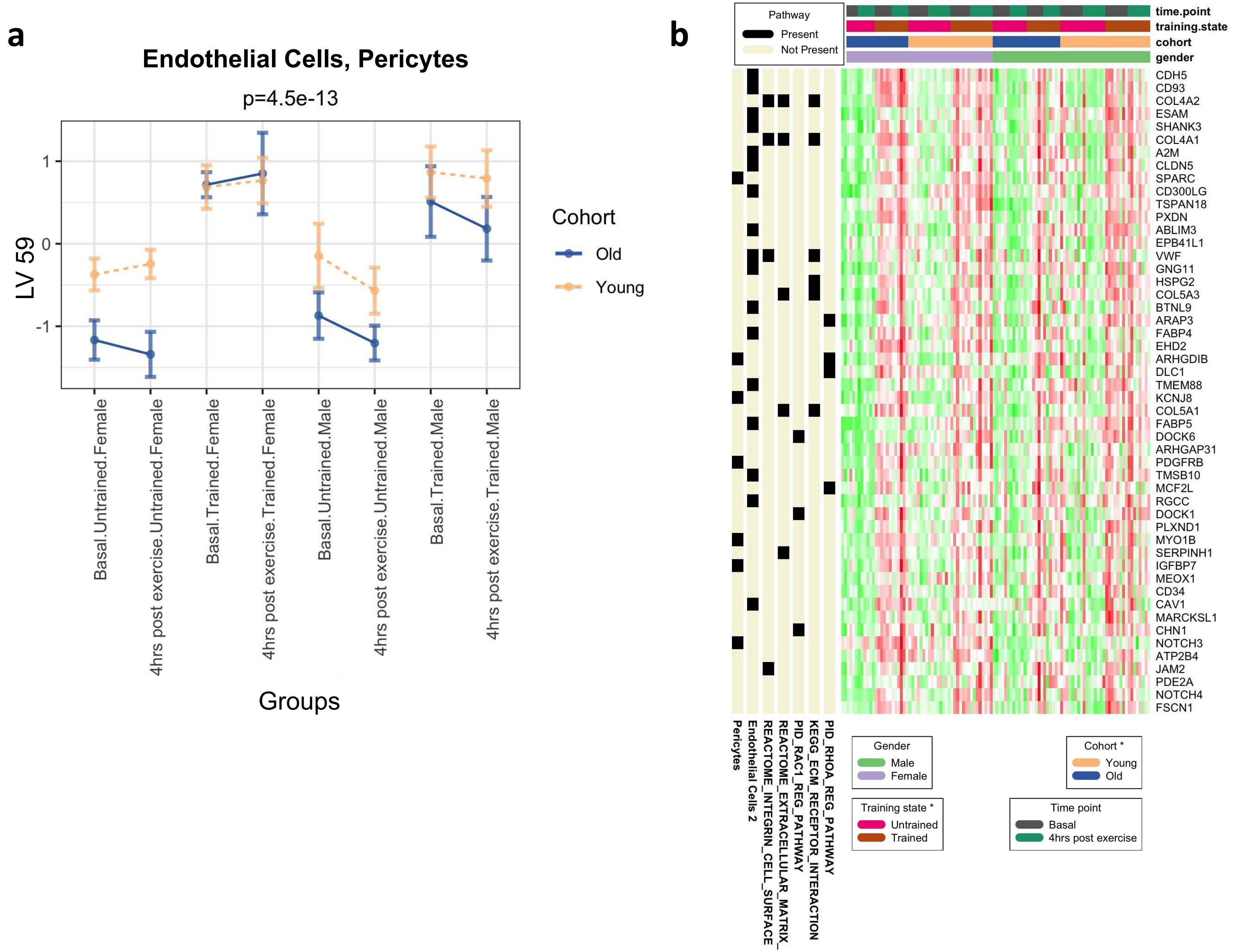

Figure S6: Endothelial cell/pericyte LV59. (A) LV59 for all groups. Points represent means of each group and error bars represent standard error. Both cohorts are depicted (orange for young and blue for old). Adjusted p-value for the four-way ANOVA is shown under the title. (B) Heatmap depicts top 50 genes for LV59. Columns represent samples and rows represent genes. Annotation rows at top show phenotypic characteristics of each sample (young vs. old, etc.) and annotation columns at left show which genesets each gene belongs to. Asterisk in legend denotes significance (adjusted $p < 0.05$) for the main effect associated with that factor in the four-way ANOVA.

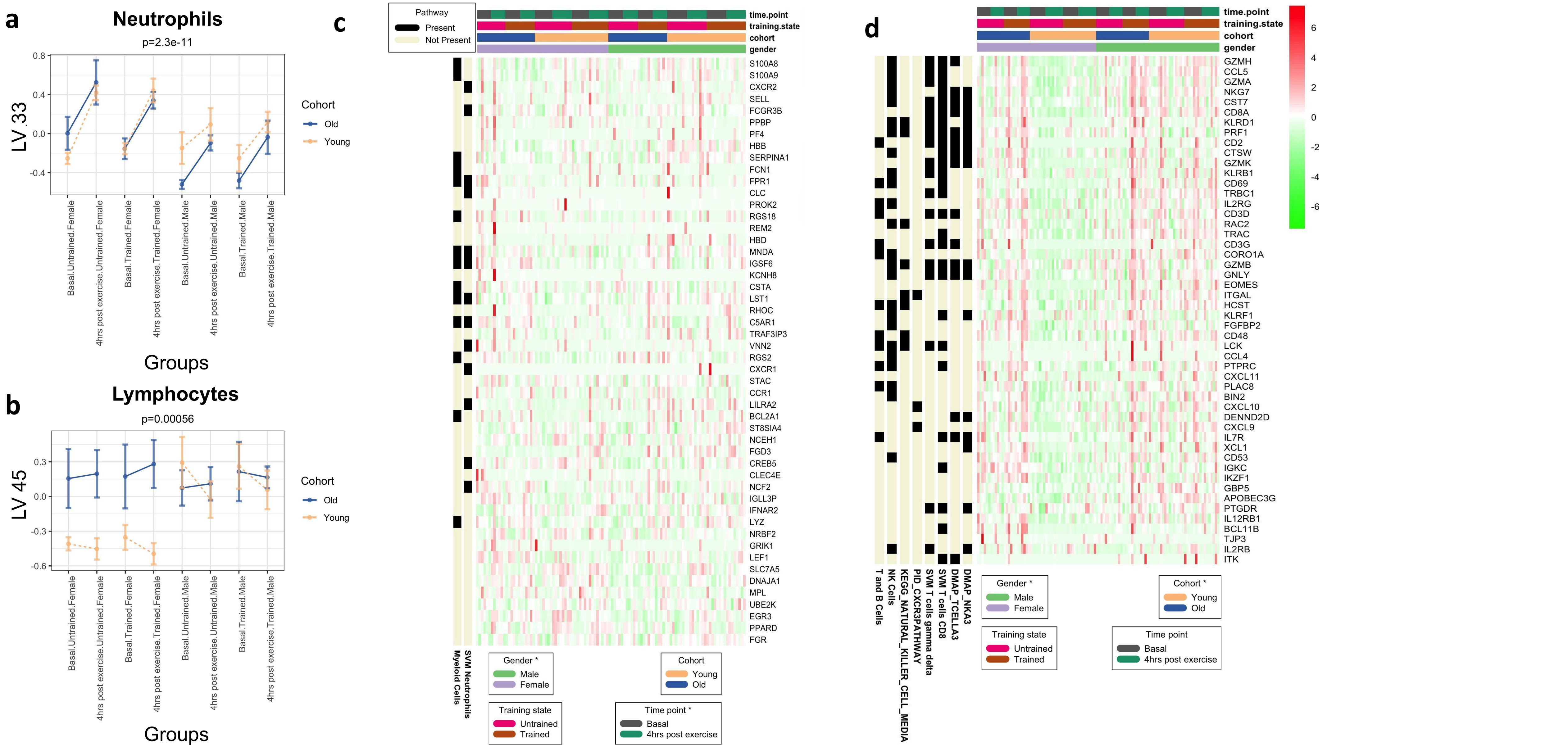

Figure S7: Myeloid cells (neutrophils) LV33 and lymphocytes LV45. (A-B) LV33 and LV45 for all groups, respectively. Points represent means of each group and error bars represent standard error. Both cohorts are depicted (orange for young and blue for old). Adjusted p-value for the four-way ANOVA is shown under each title. (C-D) Heatmaps depict top 50 genes for LV33 and LV45, respectively. Columns represent samples and rows represent genes. Annotation rows at top show phenotypic characteristics of each sample (young vs. old, etc.) and annotation columns at left show which genesets each gene belongs to. Asterisk in legend denotes significance (adjusted $p < 0.05$) for the main effect associated with that factor in the four-way ANOVA.

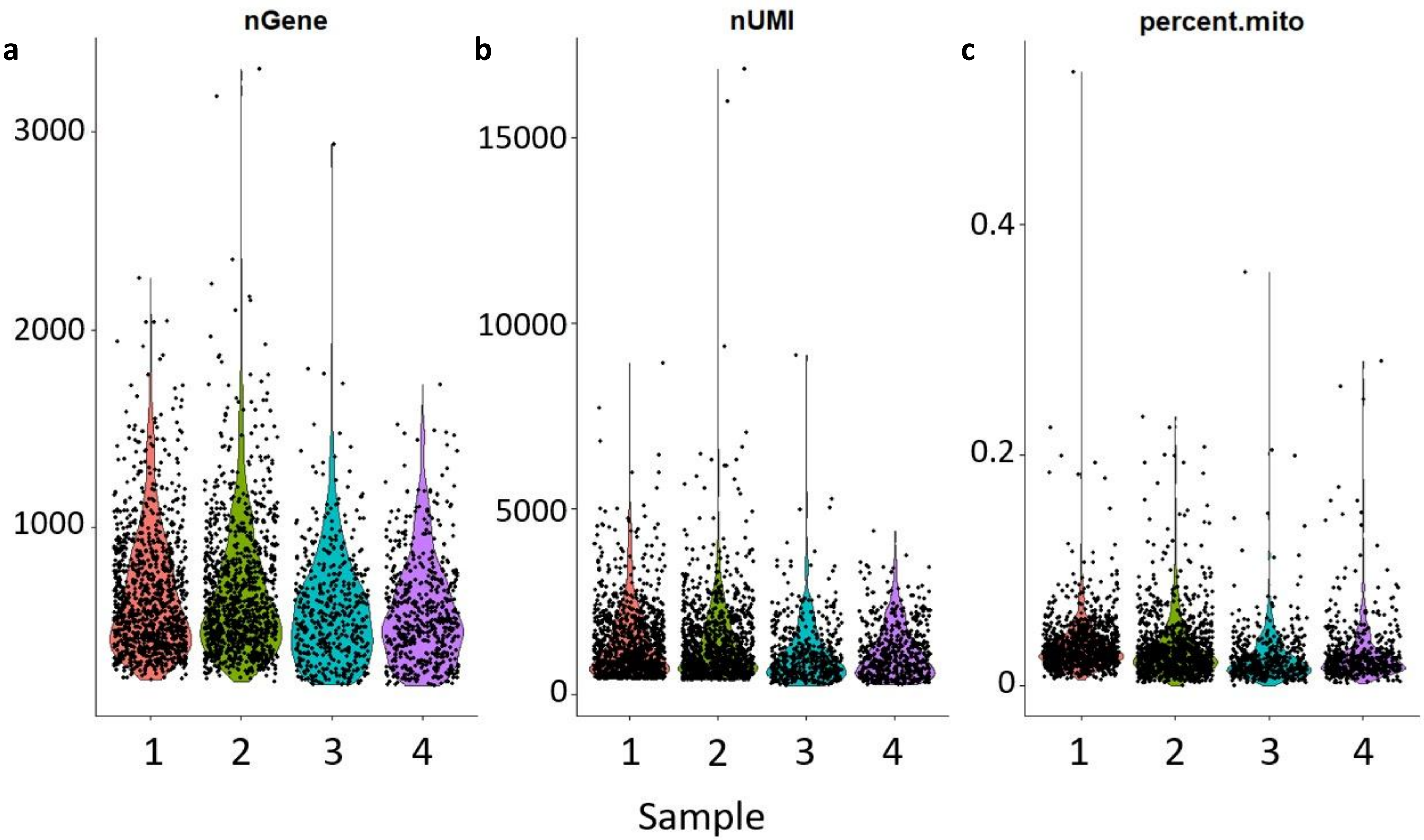

Figure S8: scRNA-seq metrics highlighting similarly good quality for each sample. (A) Violin plot of the number of expressed genes discovered per cell in each sample. (B) Violin plot of the number of unique molecular identifiers (UMIs) per cell in each sample. (C) Violin plot of the percent of UMIs from mitochondrial genes per cell in each sample.

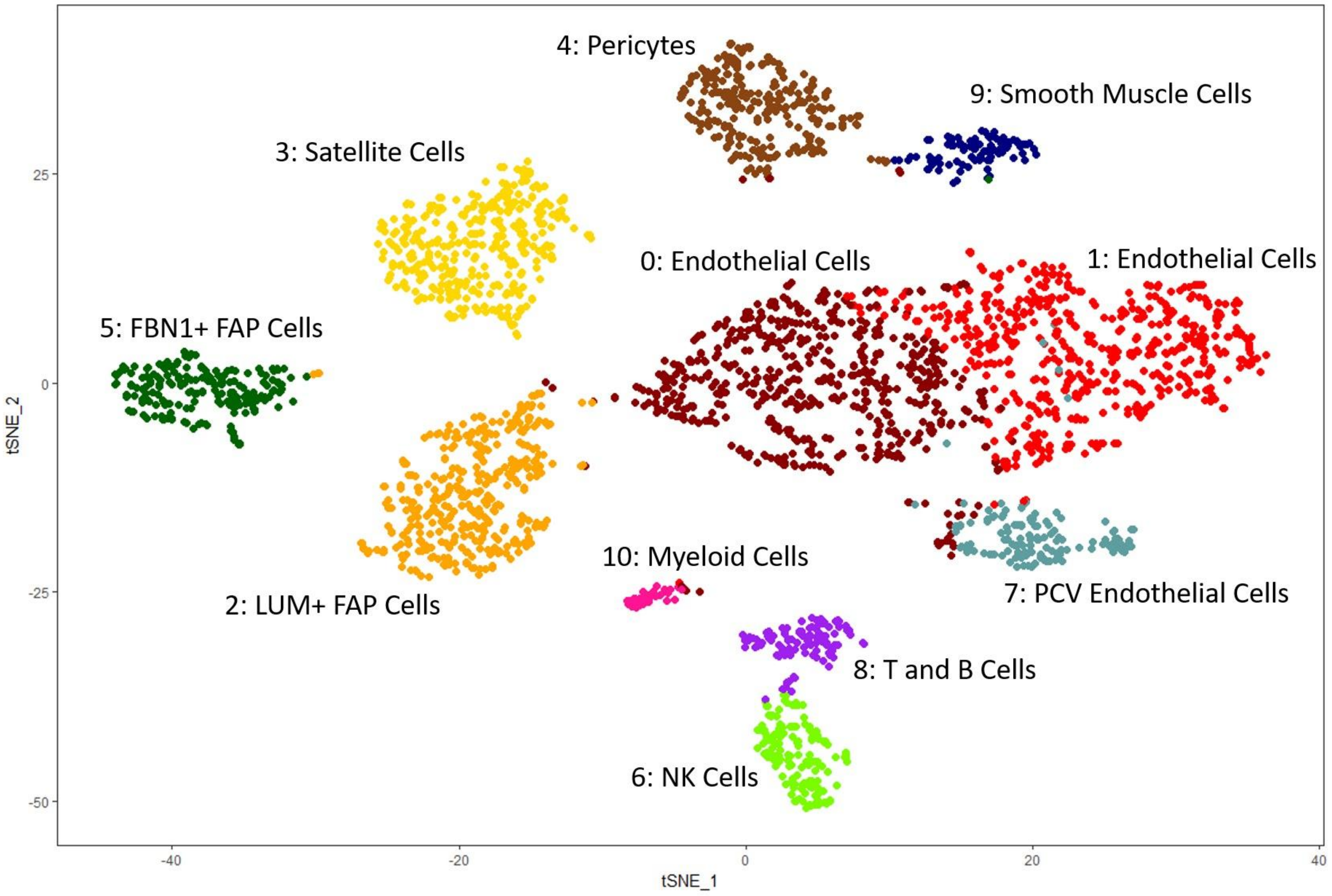

Figure S9: Cell type labeled t-SNE plot of mononuclear muscle cells from the combined set of four samples of a muscle biopsy. Cells are colored by the original clustering analysis.

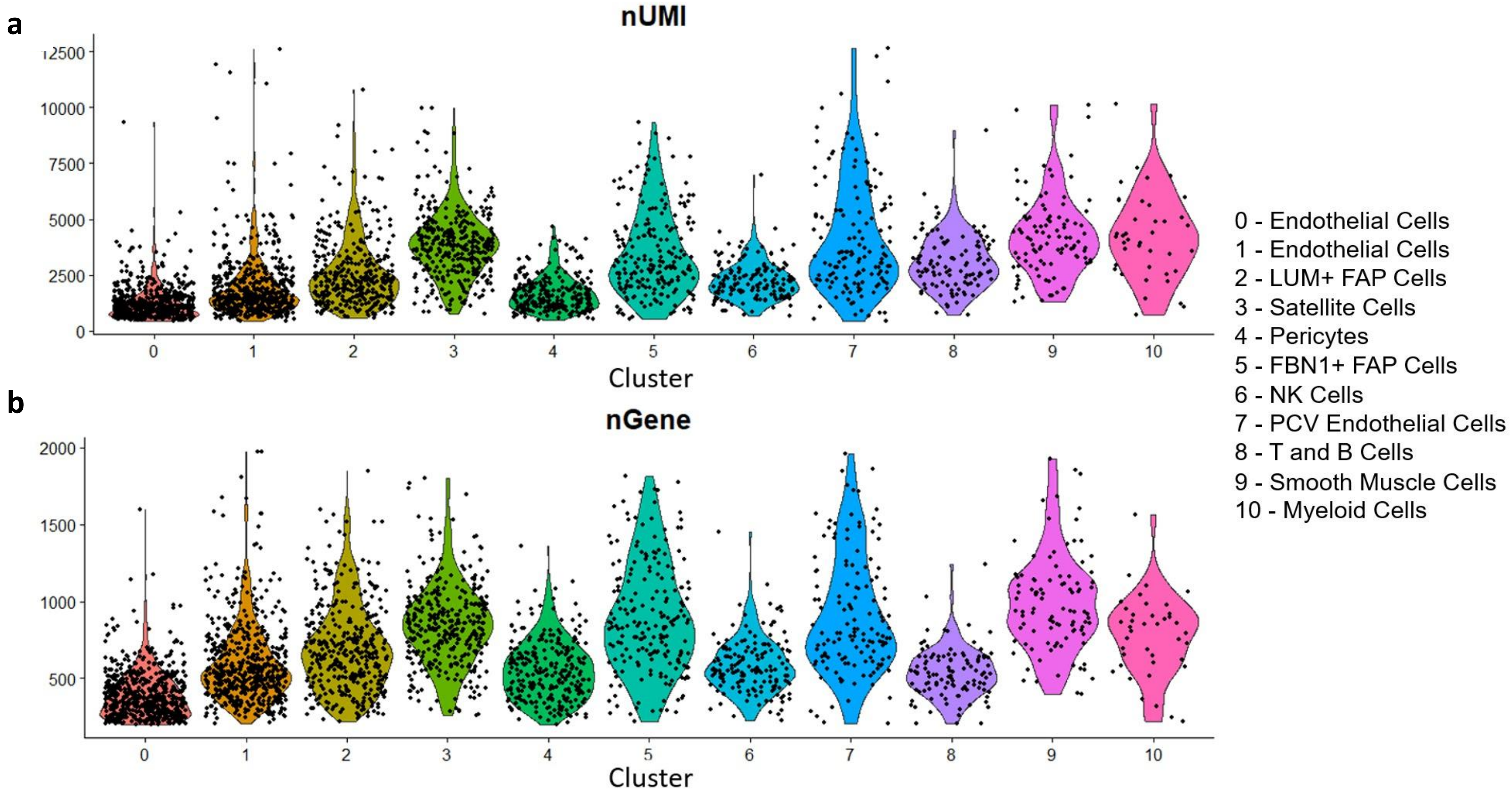

Figure S10: scRNA-seq metrics highlighting heterogeneity between clusters. (A) Violin plot of the number of UMIs per cell in each cluster. (B) Violin plot of the number of expressed genes discovered per cell in each cluster.

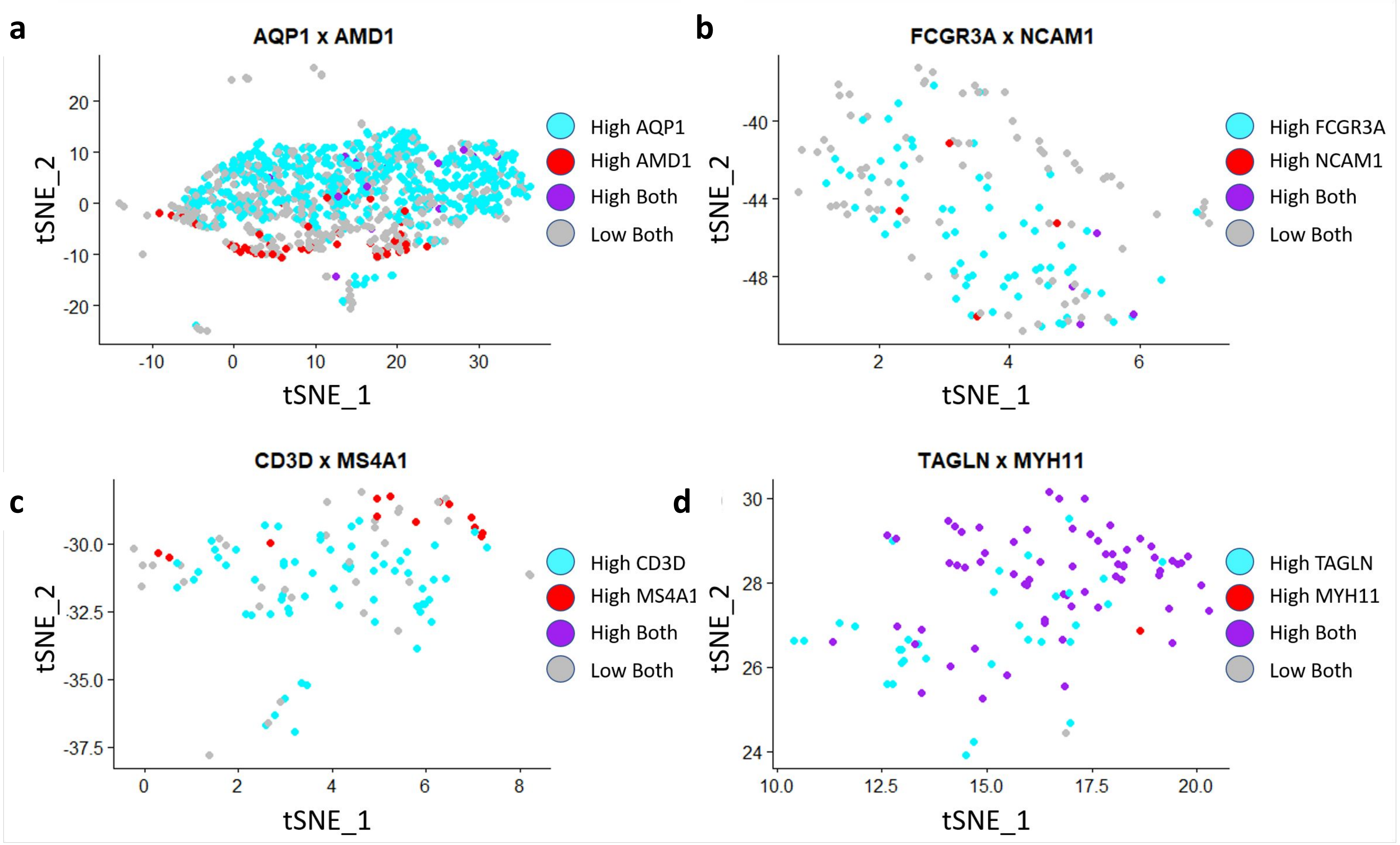

Figure S11: Differentially expressed genes within each cell type. (A) t-SNE plot of cells in the endothelial cell clusters differentiated by their expression of AQP1 (blue), AMD1 (red), or both purple. (B) t-SNE plot of cells in the NK cell cluster differentiated by their expression of FCGR3A (blue), NCAM1 (red), or both (purple). (C) t-SNE plot of cells in the T & B cell cluster differentiated by their expression of CD3D (blue), MS4A1 (red), or both (purple). (D) t-SNE plot of cells in the smooth muscle cell cluster differentiated by their expression of TAGLN (blue), MYH11 (red), or both (purple).
